# A novel LysR‐type regulator negatively affects biosynthesis of the immunosuppressant brasilicardin

**DOI:** 10.1002/elsc.202000038

**Published:** 2020-11-04

**Authors:** Marcin Wolański, Michał Krawiec, Paul N. Schwarz, Evi Stegmann, Wolfgang Wohlleben, Anina Buchmann, Harald Gross, Michael Eitel, Pierre Koch, Alma Botas, Carmen Méndez, Luz Elena Núñez, Francisco Morís, Jesus Cortés, Jolanta Zakrzewska‐Czerwińska

**Affiliations:** ^1^ Faculty of Biotechnology University of Wrocław Wrocław Poland; ^2^ Department of Microbiology and Biotechnology Interfaculty Institute of Microbiology and Infection Medicine University of Tübingen Tübingen Germany; ^3^ German Centre for Infection Research (DZIF) Partner Site Tübingen Tübingen Germany; ^4^ Department of Pharmaceutical Biology Institute of Pharmaceutical Sciences University of Tübingen Tübingen Germany; ^5^ Department of Pharmaceutical Chemistry Institute of Pharmaceutical Sciences University of Tübingen Tübingen Germany; ^6^ Departamento de Biología Funcional e Instituto Universitario de Oncología del Principado de Asturias Universidad de Oviedo Oviedo Spain; ^7^ EntreChem S.L. Oviedo Spain; ^8^ Present address: Institute of Biochemical Engineering University of Stuttgart Stuttgart Germany

**Keywords:** *Amycolatopsis japonicum*, fluorescence thermal shift, heterologous expression, *Nocardia terpenica*, secondary metabolite gene cluster

## Abstract

Brasilicardin A (BraA) is a promising immunosuppressive compound produced naturally by the pathogenic bacterium *Nocardia terpenica* IFM 0406. Heterologous host expression of brasilicardin gene cluster showed to be efficient to bypass the safety issues, low production levels and lack of genetic tools related with the use of native producer. Further improvement of production yields requires better understanding of gene expression regulation within the BraA biosynthetic gene cluster (Bra‐BGC); however, the only so far known regulator of this gene cluster is Bra12. In this study, we discovered the protein LysRNt, a novel member of the LysR‐type transcriptional regulator family, as a regulator of the Bra‐BGC. Using in vitro approaches, we identified the gene promoters which are controlled by LysRNt within the Bra‐BGC. Corresponding genes encode enzymes involved in BraA biosynthesis as well as the key Bra‐BGC regulator Bra12. Importantly, we provide in vivo evidence that LysRNt negatively affects production of brasilicardin congeners in the heterologous host *Amycolatopsis japonicum*. Finally, we demonstrate that some of the pathway related metabolites, and their chemical analogs, can interact with LysRNt which in turn affects its DNA‐binding activity.

**Abbreviations**
3‐HBA: 3‐hydroxybenzoate
BraA: brasilicardin A
Bra‐BGC: BraA biosynthetic gene cluster
BraC: brasilicardin C
BraC‐agl: brasilicardin C aglycone
BraD: brasilicardin D
BraD‐agl: brasilicardin D aglycone
CoBD: co‐inducer binding domain
DBD: DNA‐binding domain
dTmD: protein melting temperature derivative referred to control sample
EMSA: Electrophoretic mobility shift assay
FTS: Fluorescence thermal shift
GlcNAc: *N*‐acetylglucosamine
LTTR: LysR‐type transcriptional regulator
SM‐BGC: secondary metabolism biosynthetic gene clusters
TR: transcriptional regulator

## 1 INTRODUCTION

Members of genus *Nocardia* are Gram‐positive, soil‐dwelling bacteria that belong to the order of Actinomycetales. Several of these microorganisms are opportunistic human and animal pathogens that most commonly affect pulmonary tracts, cutaneous tissues and the central nervous system [1, 2]. Interestingly, besides their clinical relevance, *Nocardia* are considered, as prolific producers of medically important compounds, metabolites encoded by secondary metabolism biosynthetic gene clusters (SM‐BGCs). Up to date, numerous antimicrobial, antitumor, antioxidative and immunosuppressive substances (e.g. nocardicins, amamistatins, formobactin and brasilicardins) have already been isolated from *Nocardia* spp. (reviewed in [3, 4]). Nevertheless, the majority of nocardial SM‐BGCs are poorly or not expressed under laboratory conditions; thus, most products of these gene clusters remain cryptic or can be obtained only in small quantities. Moreover, the development of cost‐effective production of bioactive compounds from *Nocardia* species is hindered due to lack of efficient genetic tools and safety issues as most of these bacteria belong to risk group 2 microorganisms. The commonly used heterologous gene expression strategy enables activation of silent SM‐BGCs, improvement of production levels and generation of novel secondary metabolites [5, 6, 7, 8].

**FIGURE 1:**
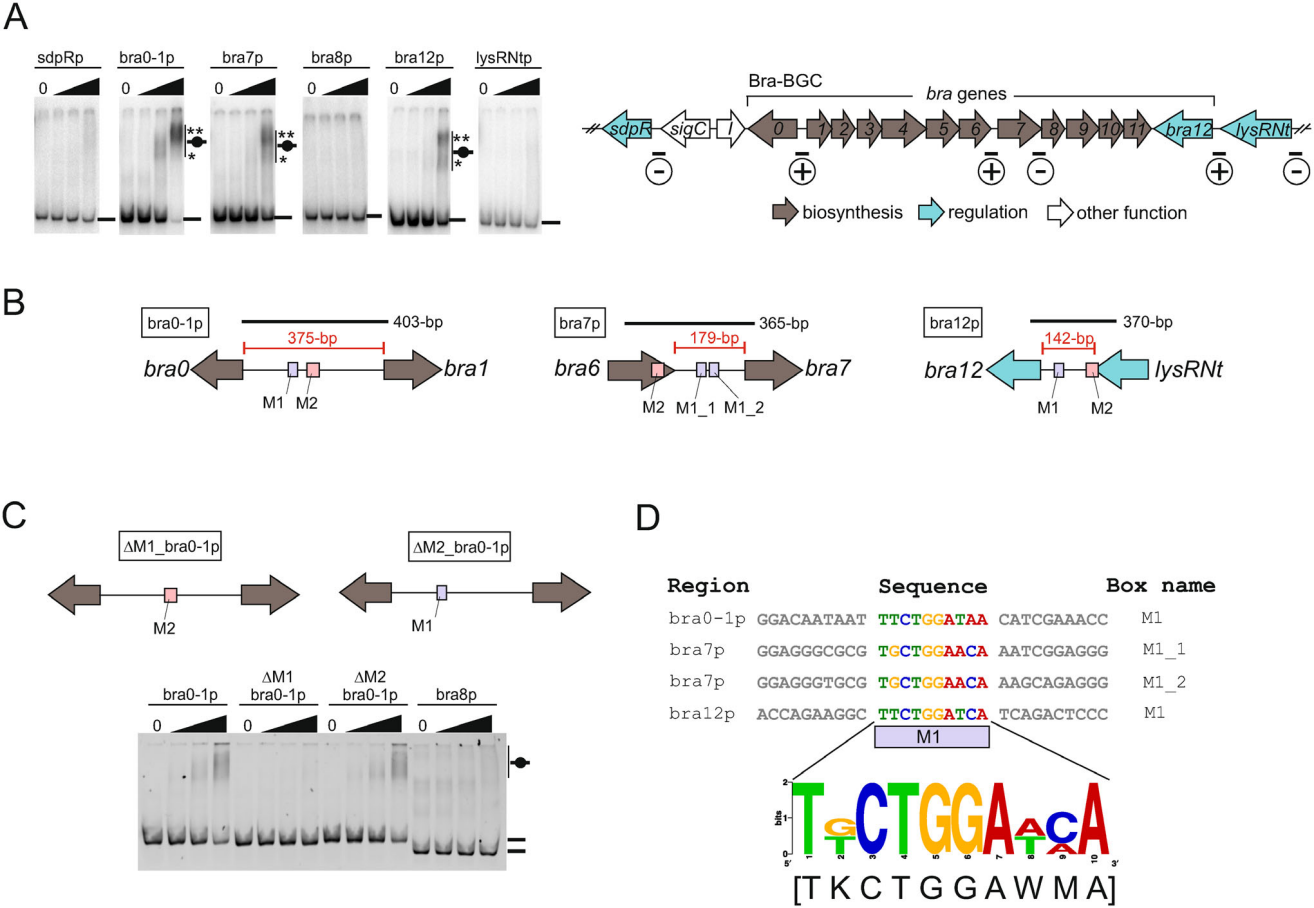
Identification of LysRNt binding sites within brasilicardin gene cluster. (A) EMSA (left panel), interaction of LysRNtHis_6_ with selected promoter regions. ^32^P radiolabeled DNA fragments were incubated with increasing concentrations of LysRNtHis_6_ (50, 250 and 500 nM ‐ black triangles) in the presence of BSA and poly(dI‐dC)·(dI‐dC) competitors. DNA‐protein complexes and unbound DNAs are marked by short black bars with and without black circle on it, respectively. The main and the additional nucleoprotein complexes are distinguished by ** and *, respectively. Graphical summary (right panel) of LysRNt binding sites within the bcaAB01 fosmid. Bound and non‐nound DNA fragments are marked with encircled “+” and “−“, respectively. (B) Schematic depiction of the in silico determined LysRNt binding sequences. The putative M1 and M2 LysRNt binding sites are shown with light violet (M1) and light red (M2) boxes (see also SI, Figure S5B). The lengths of given DNA probes (black solid lines) and intergenic regions (red solid lines) are shown. (C) EMSA, interaction of LysRNtHis_6_ with site‐specifically mutagenized *bra0‐1* promoter fragments. Cy5 fluorescently labeled DNA fragments were incubated with LysRHis_6_ protein (200, 500 and 1000 nM – black triangles) in the presence of competitors and resolved on polyacrylamide gel (bottom panel). Schematic depiction of mutagenized bra0‐1p fragments used in EMSA (upper panel, see also SI, Figure S5C). (D) Alignment of M1 LysRNt binding sequences. The corresponding sequence logo was generated using WebLogo

Brasilicardin A (BraA) is a secondary metabolite isolated from the clinical strain *Nocardia terpenica* IFM 0406 (formerly *Nocardia brasiliensis* IFM 0406) which is also known to produce the less active immunosuppressant brasilinolide A [9, 10]. BraA is a promising therapeutic compound that exhibits more potent immunosuppressive activity and lower cytotoxicity in comparison to cyclosporin A, commonly used in patients after organ transplantations [11, 12, 13].The compound is a terpenoid, which consists of a tricyclic diterpene core linked directly with an amino acid side chain and an l‐rhamnose unit and indirectly (via l‐rhamnose) with *N*‐acetylglucosamine (GlcNAc) and 3‐hydroxybenzoate (3‐HBA) moieties (Figure S6A). Besides BraA, the native strain produces also other brasilicardin congeners (BraB, BraC, BraD) (Figure S6A). However, these compounds exhibit reduced or no immunosuppressive activity in comparison with BraA [12, 14]. BraA synthesis is a multi‐step process (Figure S6B) that is largely conferred by 12 biosynthetic genes, more precisely *bra0* and divergently transcribed from it *bra1*‐*bra11* (Figure 1A). The gene cluster (*bra1*‐*bra11*) was originally identified in the Dairi lab [22], and has been recently revised to include the upstream gene *bra0* [8, 23]. All these genes, together with the *bra12* regulatory gene (introduced below) form the brasilicardin biosynthetic gene cluster (Bra‐BGC). The Bra‐BGC encodes enzymes essential for the core structure biosynthesis (*bra1‐bra6*) and methoxylation of brasilicardin skeleton (*bra0*, *bra11*), and presumably for 3‐HBA biosynthesis and attachment (*bra7‐bra9*) and l‐rhamnose attachment (*bra10*). Notably, the Bra‐BGC misses the genes involved in biosynthesis of the sugars (GlcNAc and l‐rhamnose) and GlcNAc attachment. The experimental approaches to identify genes encoding missing enzymatic activities elsewhere in *N. terpenica* genome, failed so far [8].

Transcriptional regulation within Bra‐BGC is poorly understood, and only the function of Bra12, a SARP‐like cluster situated regulator (*Streptomyces* Antibiotic Regulatory Protein) has been described so far [8]. Heterologous expression studies in an *Amycolatopsis japonicum* host demonstrated that Bra12 is an essential transcriptional regulator (TR) that activates transcription of the entire Bra‐BGC, and thus controls brasilicardin production. Consistently, overexpression of *bra12* gene, led to an increase (>60%) of the production yield of total brasilicardin congeners. However, for Bra12 neither target gene promoters within the Bra‐BGC nor the mechanism of action have yet been described. Another TR candidate for Bra‐BGC is encoded by a gene originally ascribed as ORF 11, located directly upstream of *bra12* [22]. The protein product of the corresponding gene belongs to the family of ‘LysR‐type transcriptional regulators’ (LTTRs), which are highly conserved amongst bacteria, and potentially represent the largest family of bacterial regulators (over 600,000 sequence entries in the UniProtKB database). LTTRs control an extremely broad range of cellular processes (e.g. metabolism, cell division, virulence, stress response and quorum sensing), and can respond (directly or indirectly) to various types of external signals (e.g. compounds – amino acids, aromatic compounds; light; redox changes) as reviewed [24]. Members of the LTTR family are characterized by the presence of a N‐terminal DNA‐binding domain and a C‐terminal ligand binding domain. In the classical mode of action, upon appearance of an external signal an LTTR activates transcription of genes involved in response and represses the transcription of its own gene (usually adjacent to the former one). In general, the signal modulates DNA‐binding activity of the LTTR protein to facilitate its binding to DNA and allow firing transcription of the controlled genes. However, several examples show that LTTRs may also negatively regulate expression of the response genes. In many cases, the signal is a specific ligand molecule (co‐inducer), being often a compound related with a given metabolic pathway. Binding of a ligand molecule usually triggers LTTR binding to a promoter region though in some cases it may lead to loosening the regulator from a target DNA [25, 26]. Usually, LTTRs are active as dimers or tetramers, that bind to a definite number of binding sites, most often 2–3, within a promoter region [27, 28, 15, 29]. Classical LTTR binding sequences comprise a dyad symmetry consensus T‐N_11_‐A motif; however, LTTRs’ binding sequences can significantly vary in both, the base composition and length [30, 31, 32, 33, 34].

In this work, we describe a yet unknown aspect of regulation of BraA biosynthesis. Firstly, we decipher the function of the regulator LysRNt, a novel member of LTTR family, controlling the biosynthesis of brasilicardins in *Nocardia terpenica* IFM 0406. Finally, we analyze the potential ability of compounds related with the brasilicardin biosynthetic pathway to modulate activity of this regulator.

## 2 MATERIALS AND METHODS

### 2.1 Bacterial strains, culture conditions

Strains used in this study are listed in Table S1 in Supplementary Information (SI). For *Escherichia coli*, the culture conditions, media, antibiotic concentrations followed general protocols [35] except for recombinant protein expression (see below). For *Amycolatopsis japonicum* strains, the culture growth conditions and media followed the procedures described previously [8].

### 2.2 DNA manipulations, plasmid and strain construction

Standard molecular biology procedures were used [35, 36]; for plasmid construction the sequence and ligation‐independent cloning was used [37]. Plasmids and PCR products were purified using commercial kits (ThermoFisher Scientific and A&A Biotechnology). Chemically competent *E. coli* DH5α were prepared and transformed according to standard protocols. Plasmid constructs were verified by DNA sequencing. For plasmid introduction into *A. japonicum* the intergeneric conjugation procedure with modifications was used [8, 36]. Plasmid and strain construction are described in the SI. Plasmids, fosmids and oligonucleotides (supplied by Merck) used in the study are listed in Tables S1 and S2. Enzymes used in this study were supplied by ThermoFisher Scientific and New England Biolabs.

**PRACTICAL APPLICATION**

Brasilicardin A is a promising powerful immunosuppressant produced naturally by a pathogenic bacterium *Nocardia terpenica*. However, its production in biological systems is limited to either low yield of the final compound in the native host or incomplete biosynthesis in heterologous hosts. This report describes yet unknown aspect of regulation of brasilicardin biosynthesis by the negative cluster situated regulator LysRNt. This study can have further impact on the improvement of brasilicardin congeners production yields.

### 2.3 Protein LysRNtHis_6_ purification

A detailed procedure was described in the SI. Briefly, the *E. coli* Rosetta™ 2(DE3) harboring the expression pET‐21a(+)lysRNt plasmid, carrying *lysRNt* gene, was used to produce C‐terminally His‐tagged LysRNt (LysRNtHis_6_). The LysRNtHis_6_ was purified by metal affinity chromatography (Talon column) using an Äkta Start system (GE Healthcare). To assess protein purity, samples were resolved using sodium dodecyl sulfate‐polyacrylamide gel electrophoresis (SDS‐PAGE) [38] and stained with PageBlue™ protein staining solution (ThermoFisher Scientific). To examine LysRNtHis_6_ for oligomerization the approach described previously with minor modifications was used [39].

### 2.4 Electrophoretic mobility shift assay (EMSA)

The EMSA used to analyze DNA‐protein interactions was performed similarly as described previously [39] – described in detail in the SI. Briefly, purified LysRNtHis_6_ protein at different concentrations was incubated with a radiolabeled (^32^P) DNA fragment (∼500 bps), in 1x phosphate‐buffered saline (PBS) buffer supplemented if necessary with BSA, glycerol and non‐specific competitor poly(dI‐dC)·(dI‐dC). In respective reactions, the small‐compounds were added to the complete reaction mixtures at the initial stage of incubation. The reactions were resolved on either agarose or polyacrylamide gels prepared in trisborate‐EDTA (TBE) and then visualized using autoradiography. Band intensities were calculated using Gel Analyzer 2010 software.

### 2.5 Detection of brasilicardin congeners

To detect production of brasilicardins in *A. japonicum* strains, the procedure described previously was applied [8]. Briefly, a preculture grown in tryptic soy broth medium (TSB, for 48 h) was transferred into SM17 medium (1:100 ratio) and cultivated for 72 h. The cultures were then centrifuged, and the supernatants were used directly for HPLC/MS analysis. To calculate total brasilicardin production, HPLC/MS intensities of each brasilicardin congener (BraC, BraC aglycone, BraD and BraD aglycone) were summed up.

### 2.6 Fluorescence thermal shift (FTS)

The FTS assay followed generally described protocols and manufacturer recommendations for the StepOnePlus™ instrument. The FTS reaction mixture (20 μL) contained: purified LysRNtHis_6_ (2 μM), 1x phosphate‐buffered saline (PBS) supplemented with glycerol (5%), SYPRO® Orange dye (Merck) (5x) and compound at the given concentration. The compounds used in the assay were first dissolved in 50% ethanol and subsequently added to the reaction mixture to obtain the desired concentrations; the final ethanol concentration in all reaction mixtures was 0.1%. After incubation at room temperature (RT; ∼25°C) (10 min) the samples were transferred to a StepOnePlus™ Real‐Time PCR System employing the following parameters: experiment type – Melt Curve, reporter dye – ROX, quencher dye – none; heating parameters: step 1 – incubation at 25°C for 10 min; step 2 – 99°C 2 min; ramp rate parameters: step 1 – 100%, step 2 – 1%. The analyses of protein melt curve plots and calculations of delta melting temperature derivatives (dTmD) were conducted using the Protein Thermal Shift Software 1.3 (ThermoFisher Scientific). The graphs and p‐value calculations were obtained using RStudio package. The hit threshold settings for dTmD were adopted from the Protein Thermal Shift Software, dTmD > 0.2°C.

### 2.7 Bioinformatics tools, nucleotide and amino acid sequences

The web resources used in the study are listed in Table S4. Briefly, the following tools were used: identification of protein domains – SMART (normal mode) [40, 41]; determination of protein secondary structure – PSIPRED [42, 43]; generation of alignment graphs – UGENE [44]; searches for protein homologs – BLASTp (protein‐protein blast) and Phyre [45]; in silico prediction of protein DNA binding sites – MAST‐MEME (Motif Alignment & Search Tool) of MEME suite package [46] (search parameters were as follows: discriminative mode; maximum 3 different sequence motifs; occurrence 0 or 1 per sequence; motif length range 6 to 50 nucleotides); generation of sequence logos – WebLogo [47]; additional DNA motif searches ‐ the Pattern Locator [48]. The *lysRNt* nucleotide sequence and LysRNt amino acid sequences are available on the NCBI under AWN90_RS33340 and WP_067591001.1 numbers, respectively. The master record for *Nocardia terpenica* IFM 0406 genome sequence and the complete sequence of the Bra_BGC are available under GenBank numbers LWGR00000000.1 [23] and MT247069, respectively.

## 3 RESULTS

### 3.1 In silico analysis identifies LysRNt a novel member of LysR‐type transcriptional regulators

**FIGURE 2:**
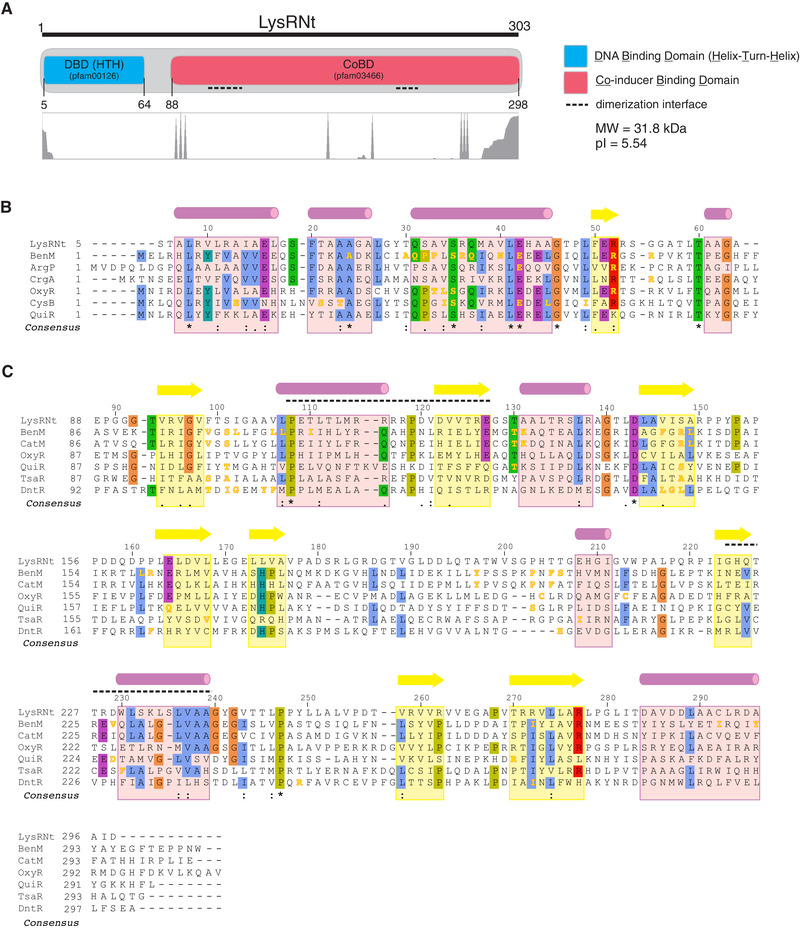
LysRNt domain architecture and sequence alignments. (A) Domains were identified using SMART webtool. The DNA (DBD) and co‐inducer (CoBD) binding domains and corresponding protein family identifiers (pfam) together with theoretical pI and molecular weight (MW) are shown. Below, a graphical representation (area graph in gaps mode) of the multiple sequence alignment of LysRNt and its 100 top homologues from NCBI BLASTp search. Multiple sequence alignments (B) and (C), for DBD (amino acids 5–64) and CoBD (amino acids 88–298) domains, respectively. The alignments were created using Ugene software (ClustalW algorithm). The consensus sequences (shown on the bottom) and residues color shadings are in ClustalW format (symbols: asterisk ‐ positions with fully conserved residue, colon ‐ conservation between groups of strongly similar properties, period ‐ conservation between groups of weakly similar properties; conserved residue shading are: blue – hydrophobic, red – positively charged, magenta – negatively charged, green – polar, orange – glycines, yellow – prolines, cyan – aromatic, white ‐ unconserved). Secondary structure elements, alpha‐helices and beta‐strands, predicted using the PsiPred webtool, are shown as pink cylinders and yellow arrows, respectively. Numbering shown on top reflects amino acid positions in LysRNt sequence. (B) The protein designations, UniProt identifiers and references are: BenM (O68014) [15]; ArgP (P9WMF5) [16]; CrgA (Q9JXW7); OxyR (P0ACQ4); CysB (P06614); QuiR (Q8Y9N7). Residues playing key functions in motif recognition (BenM) and interactions with DNA (ArgP, CrgA, OxyR, CysB) are shown in yellow. (C) The protein designations, UniProt and PDB identifiers, ligands and references are: BenM (O68014; 2F78, benzoate and muconate) (residues interacting with benzoate and muconate has been indicated; note that residues interacting with muconate are conservative between BenM and CatM and thus for clarity have been indicated on CatM sequence) [17]; CatM (P07774; 2F7C; muconate) [17]; OxyR (P0ACQ4; 1I69 and 1I6A; no ligand) – C199 and C208 form disulfide bridge [18]; QuiR (Q8Y9N7; 5TED; shikimate) [19]; TsaR (P94678; 3N6U; p‐toluenesulfonate) (not published); DntR (Q7WT50; 1UTB; salicylate) [20, 21]

The brasilicardin biosynthetic gene cluster (Bra‐BGC) contains 13 genes (*bra0*‐*bra11, bra12*) involved in the biosynthesis of BraA, amongst which *bra12* encodes an essential for brasilicardins production TR, Bra12. Looking for other putative TRs of the Bra‐BGC, we analyzed the sequence of bcaAB01 fosmid (2,712,512‐2,752,696 nucleotides on scaffold2; accession KV411304.1) comprising the entire Bra‐BGC and the flanking genes [8]. Within the selected region, in addition to *bra12*, we have identified four other genes encoding putative DNA‐binding regulators: KstR, LysRNt, OmpR, SdpR (Table S3). In this study, we address the LysRNt protein encoded by a gene under locus tag no. AWN90_RS33340, originally ascribed as ORF11 [22] (hereafter, *lysRNt*). LysRNt belongs to a large and conserved across bacteria family of ‘LysR‐type transcriptional regulators’ (LTTRs) (Figures 2A and S1). Amino acid sequence analysis (BLASTp on NCBI) demonstrated that LysRNt displays the highest identity (>50% for query coverage >90%) to LTTRs of high GC‐rich Actinobacteria (e.g. *Nocardiaceae* and *Streptomycetaceae* families) and relatively high identity (>30%) given to LTTRs of other distant bacterial classes (e.g. α‐Protobacteria). A BLASTp search against the *N. terpenica* reference proteome (Proteom ID: up000076512) indicated lack of LysRNt homologues (>50% identity for query coverage >90%) (data not shown). A SMART [41] search for functional protein domains indicated that LysRNt, like other LTTRs, comprises two domains (Figure 2A), a relatively well conserved N‐terminal DNA‐binding domain (DBD) (amino acids 5–64) and a less conserved C‐terminal ligand binding domain (amino acids 88–298) referred often to as co‐inducer binding domain (CoBD).

In silico analysis indicates that the putative DBD of LysRNt (LysRNt_DBD) contains common for TRs helix‐turn‐helix (HTH) motif often conferring DNA binding activity. The HTH motif of LysRNt is classified into the HTH_1 protein domain family‐ (pfam00126), characteristic for LysR‐family regulators (Figure 2A). LysRNt_DBD exhibits full or high amino acid conservation at many positions within the DBD domains of other well studied LTTRs (e.g. BenM, ArgP, CrgA, OxyR and QuiR) (Figure 2B), involved for instance in aromatic compounds degradation, amino acid transport, regulation of chromosome replication and response to oxidative stress. Some of these conserved amino acids seem to play an essential function in DNA binding (yellow letters in Figure 2B) [15, 16, 49–19, 51]. Though, despite the high sequence conservation, in most cases these LTTRs exhibit different binding sequence specificities.

The C‐terminal CoBD (LysRNt_CoBD) constituting presumably a sensory part of the protein shares similarity to the conserved LysR_substrate protein domain family (pfam03466), a member of the type 2 periplasmic proteins (PBP2) superfamily (cl25412). This family includes membrane transporters, receptors and TRs exhibiting broad range of ligand specificity.[52, 53] In contrast to DBD, the overall amino acid sequence identity of LysRNt_CoBD is relatively low among LTTR members (identity range 20 to 30%) with only a few amino acids fully conserved among all sequences (Figure 2C). In‐depth sequence analysis indicated that LysRNt_CoBD shares a few amino acids that have been reported to be involved in interactions during binding of small aromatic compounds (e.g. salicylate structural analogs) in other LTTR members (yellow letters in Figure 2C) (for details see subsequent sections). The CoBDs also play crucial roles in LTTRs dimerization. Sequence analysis of LysRNt_CoBD (NCBI BLASTp > Conserved Domains) revealed the presence of a presumable dimerization interface (amino acids 108–127 and 224–239), resembling the conserved interface present in the LysR regulators (Figures 2C and S2) (see also Section 3.2).

### 3.2 LysRNt binds biosynthetic and regulatory gene promoters within the brasilicardin gene cluster

To conduct in vitro analyses, we first purified a recombinant, C‐terminally His‐tagged version of LysRNt (LysRNtHis_6_; 309 aa, 32.6 kDa, pI 6.04) using the common pET system. The protein was expressed from the pET‐21a(+)lysRNt plasmid and purified using metal affinity chromatography (purity ca. 95% as judged by SDS‐PAGE) (Figure S3AB). Our few first attempts to overexpress the target protein were unsuccessful, resulting in the detection of only trace amounts of the His‐tagged protein when using anti‐HIS antibody and Western‐blot analysis (Figure S4A‐C). Investigation of the genome nucleotide sequence revealed that the expression construct (pET‐21a(+)lysRlnNt) used in those experiments carried a mis‐annotated (derived from the preliminary genome annotation stage) version of the *lysRNt* gene (*lysRlnNt*) encoding a N‐terminally extended and probably highly unstable recombinant protein (LysRlnNtHis_6_) (see SI). Indeed, analysis of the re‐annotated genome of *N. terpenica* confirmed this assumption [23], and allowed construction of a proper expression plasmid, as described above. Since most of LTTRs tend to form oligomers, we also tested the LysRNtHis_6_ for the ability to oligomerize. However, our approaches exploiting an in vitro glutaraldehyde crosslinking method in combination with SDS‐PAGE and Western‐blot analyses, did not prove LysRNtHis_6_ dimer‐ or oligomerization and solely a monomeric form was observed (Figure S3C). Similar effects were obtained with the use of different than glutaraldehyde crosslinking agents (data not shown).

To examine whether the LysRNt could potentially control the genes within Bra‐BGC, we sought to identify the promoter regions that are bound by LysRNtHis_6_ ‐ for this purpose we used EMSA. Based on gene function and their organization within the Bra‐BGC, we selected several upstream regions of the genes encoding enzymes essential for brasilicardin biosynthesis (intergenic *bra0*‐*bra1*) and confirmed/putative TRs (*bra12*/*sdpR, lysRNt*, respectively). In addition, since the Bra‐BGC is probably organized in sub‐operons (Figure S5A) another putative intergenic promoter region (*bra7* upstream sequence) as well as an intragenic negative control fragment (*bra8* upstream sequence) has been chosen for analysis (Figure 1A).

In EMSA experiments, we observed specific binding of LysRNtHis_6_ to DNA fragments encompassing *bra7*, *bra12* and intergenic *bra0‐bra1* gene promoter regions (bra0‐1p, bra7p and bra12p) and did not observe retarded migration of the DNA fragments harboring *sdpR*, *bra8* and *lysRNt* gene promoters (sdpRp, bra8p and lysRNtp) (Figure 1A). The DNA shifts were observed only at relatively high LysRNtHis_6_ concentrations (250 and 500 nM) and exhibited a diffused appearance, that may suggest unstable protein binding to DNA, probably due to low LysRNt binding affinity to its DNA targets. We also observed formation of double nucleoprotein complexes for each of the retarded fragments, the main one (higher molecular weight) and the faint one (lower molecular weight) migrating below (depicted by ** and *, respectively) that may reflect gradual protein binding to relaxed DNA sequences at higher protein concentrations. At the highest concentration LysRNtHis_6_ (500 nM) bound nearly all available bra0‐1p DNA, and significantly smaller portions of bra7p and bra12p DNAs, indicating that the former fragment is bound with highest affinity amongst the tested promoter regions.

Trying to precisely determine LysRNt binding sites, we applied DNaseI footprinting. However, we did not observe the assay‐typical protection patterns (data not shown), probably due to low DNA binding affinity of LysRNt and/or due to the unstable nature of a LysRNt‐DNA interaction. Thus, to predict putative LysRNt binding sequence within the bound promoter regions, we applied a *de novo* motif discovery employing MEME Suite tool [46]. The MAST‐MEME results revealed two probable sequence motifs (binding sites), M1 and M2, common to all three bound promoter regions and absent from non‐bound fragments (Figure 1B and Figure S5B). The bra0‐1p and bra12p contain single copies of M1 and M2 motifs, while two exact copies of M1 box (M1_1 and M1_2) have been recognized in *bra7* upstream region. To verify LysRNt binding to the in silico predicted sequences, we conducted additional EMSA analysis with the DNA fragments harboring mutated M1 or M2 motifs (Figure 5SC). In this experiment, LysRNtHis_6_ interacted with the bra0‐1p (wild‐type sequence) and ΔM2_bra0‐1p (M2 mutant) fragments but did not interact with the ΔM1_bra0‐1p fragment containing mutated M1 site (Figure 1C), indicating that M1 motif comprises the binding sequence of LysRNt.

The M1 motif is described by 5′‐3′ consensuses TKCTGGAWMA (T‐N8‐A) and lack typical for many LTTRs binding sites dyad sequence symmetry (Figure 1D). Likewise, the classic LTTR T‐N_11_‐A DNA‐binding motif [32, 33, 54], the M1 sequence possesses T and A nucleotides in the flanking positions. In sum, we have demonstrated that LysRNtHis_6_ interacts with promoter regions of crucial genes directly involved in brasilicardin biosynthesis (*bra0, bra1*, *bra7*, and *bra12*). The LysRNt binds also the *bra7* upstream region suggesting possible regulation of the downstream *bra7*‐*bra11* genes.

### 3.3 Pathway specific compounds and their analogs modulate LysRNt activity in vitro

Many LTTRs bind ligands that can modulate their DNA binding activities. As shown by sequence analysis the LysRNt comprises few conservative amino acids that could putatively be involved in binding salicylic acid analogs. To investigate possible binding of the compounds related with brasilicardin biosynthetic pathway to the LysRNt protein and their influence on the regulator's interaction with DNA, we applied FTS and EMSA, respectively.

**FIGURE 3:**
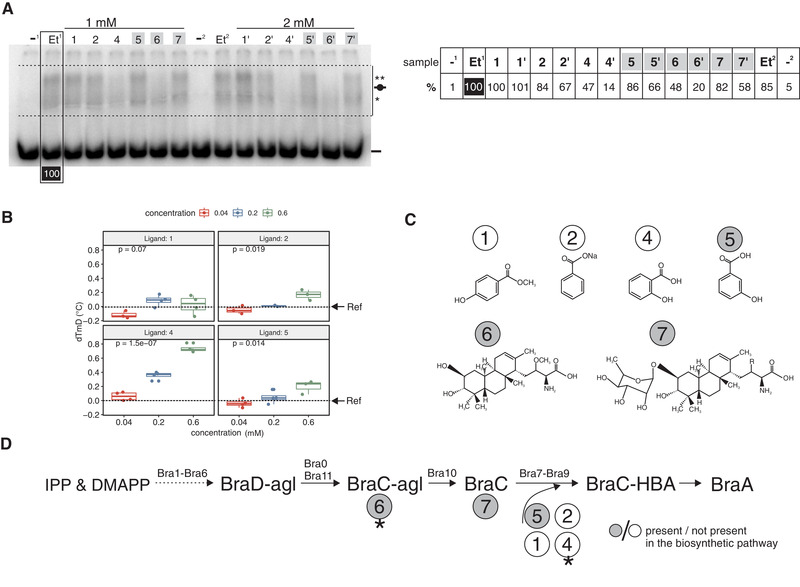
Regulation of LysRNt activity by the small‐compounds. (A) (Left panel) EMSA, interaction of LysRNtHis_6_ (500 nM) with ^32^P labelled bra12p DNA in the presence of the small‐compounds: 1, 2, 4, 5, A, C at 1 and 2 mM concentrations (2 mM samples are marked with an apostrophe). The controls (Et^1^ and Et^2^) contained EtOH (1%) instead of dissolved compounds; the protein‐free reactions (‐^1^ and ‐^2^) contained EtOH (1%) and lacked LysRNt. DNA‐protein and unbound DNAs are marked by short black lines with and without black circle on it, respectively. The main and the additional nucleoprotein complexes are distinguished by ** and *, respectively. The Et^1^ sample was used as a reference sample (100% intensity). The gel section used for calculations of DNA intensity is limited by dotted lines. The samples containing metabolites of the corresponding biosynthetic pathway are highlighted with grey background. (Right panel) A summary of the DNA bound in the DNA‐protein complexes expressed as a percentage of intensity measured for control sample (Et^1^, 100%). (B) FTS study, interaction of LysRNtHis_6_ with the small‐compounds. The delta of melting temperature derivatives (dTmD) were measured for compounds 1, 2, 4, and 5 at three concentrations (0.04, 0.2, and 0.6 mM). The LysRNt protein (2 μM) incubated with solvent EtOH (0.1%) was used as reference sample (Ref; dTmD = 0) and is schematically marked with a short arrow and horizontal dotted line. The box plot represents results of at least three technical measurements performed for each compound concentration. The software default positive hit threshold was 0.2°C > dTmD. The graph was obtained using RStudio software. (C) Structures of compounds used in EMSA and FTS studies: 1 = methyl 4‐hydroxybenzoate, 2 = sodium benzoate, 4 = salicylic acid, 5 = 3‐hydroxybenzoic acid, 6 = BraC‐aglycone, 7 = BraC. (D) BraA biosynthetic pathway (based on [8]). The enzymes (Bra0‐Bra11) are listed above arrows, the abbreviated names of compounds utilized or synthesized in enzymatic reactions are given. Asterisks (*) indicate compounds that influenced most significantly the LysRNt DNA binding activity. BraC‐HBA – BraC‐3‐hydroxybenzoic acid; BraC‐agl – BraC‐aglycone; BraD‐agl – BraD aglycone; DMAPP – dimethylallyl diphosphate; IPP – isopentenyl diphosphate

For these experiments, we selected a set of compounds, collectively referred to as small‐compounds. These included pathway metabolite 3‐HBA, also referred as **5**, salicylic acid (**4**) and its two structural analogs methyl hydroxybenzoate and sodium benzoate (designated as **1** and **2**), and brasilicardin congeners, BraC‐aglycone (**6**, also referred as BraC‐agl) and BraC (**7**) (Figure 3C,D).

To study the effect of small‐compounds on LysRNt‐DNA complex formation, we used a bra12p fragment that exhibited intermediate binding to LysRNtHis_6_ at 500 nM (Figure 1A); thus, allowing observations of changes in protein‐DNA complexes abundance. To check for dose dependence, we tested all compounds at two concentrations (1 and 2 mM). The analyses revealed that the formation of nucleoprotein complexes (LysRNtHis_6_‐bra12p) was negatively affected in the presence of all, except **1**, small‐compounds with the most pronounced effect observed for compounds **6** and **4** (Figure 3A). As compared to the control compound‐free sample (Et^1^), in the presence of **6** and **4** (1 mM), less than half amounts of corresponding protein‐DNA complexes remained in the lanes (48 and 47%, respectively), indicating that both compounds effectively diminished LysRNt binding affinity to its target DNA. The negative effects exerted by other compounds, such as the pathway related **7** and **5**, and pathway unrelated **2** were considerably weaker (82, 86 and 84%, respectively, at 1 mM) or none for compound **1**. In all cases, except for **1**, the decrease in abundances of nucleoprotein complexes was more pronounced at 2 mM of given compound indicating that the effect was concentration dependent. Supporting EMSA experiments performed in the presence of the crosslinking reagent (added to prevent destruction of a fragile nucleoprotein complex during electrophoresis) delivered, in most cases, similar results (Figure S7AB). In addition, detailed inspection of nucleoprotein bands revealed in some cases (most clearly visible for samples **6** and **6′** in Figure 3A) the preferential disassembly of the upper main nucleoprotein band (**) in the presence of the compounds.

In order to analyze the small‐compounds interactions with LysRNt, we applied FTS. Ligand binding to its protein usually stabilizes and results in the increase of the protein's melting temperature (Tm) monitored by the changes of fluorescence of fluorescent dye associated with the protein [53, 55]. Initial screening demonstrated that recombinant LysRNt (2 μM) produced typical melt curves acceptable in FTS analyses (Figure S7C). However, these tests also revealed that, for unclear reasons, the compound BraC‐aglycone and BraC significantly quenched the fluorescence produced in the assay making them impossible for use in further analyses (data not shown). Thus, only 3‐HBA (**5**) and its analogs **1**, **2** and **4** were used in subsequent experiments. These analyses revealed that pathway unrelated salicylic acid (**4**), induced the highest increase of LysRNtHis_6_ melting temperature with the dTmD (the Tm derivative referred to the control sample) values 0.35 and 0.74°C (at 0.2 and 0.6 mM concentrations, respectively) (Figure 3B). The brasilicardin pathway metabolite 3‐HBA (**5**) and the pathway unrelated compound sodium benzoate (**2**) protein exerted much weaker stabilizing effect with dTmD values slightly below the set threshold (0.17 and 0.19°C, respectively, at 0.6 mM), and there was nearly no effect on Tm in the presence of methyl hydroxybenzoate (**1**) (dTmD = 0.02°C at 0.6 mM).

To get additional insight into aromatic ligands recognition by LysRNt, we analyzed in detail the similarity of LysRNt_CoBD to CoBDs of other LTTRs binding that type of ligands selected from the sequences deposited in the protein data base (PDB) and the protein structure prediction server Phyre2. In the alignment four regulators BenM, QuiR, TsaR, DntR binding benzoate, shikimate, toluenesulfonate and salicylate, respectively (Figures 3C and S6C), were used. We also included the well described model OxyR protein, which responds to the redox signals by changing the status of its disulfide bridges. The alignment indicated that none of the few fully conserved amino acids (P108, D143, P247 – positions for LysRNt), have been previously reported to be involved in interactions with ligands (yellow letters Figure 2C). However, LysRNt shares with examined LTTRs several other less or non‐conserved residues (S101, L107, T130, P203, D229) contributing to interactions with ligands in these proteins. For example, L105 of BenM, equivalent of L107 in LysRNt, was demonstrated to interact with benzoate [17]; T130 of LysRNt is preserved in four analyzed regulators and in QuiR it plays a role in shikimate recognition [19].

In sum, we have demonstrated that small‐compounds can negatively influence LysRNtHis_6_ binding to DNA (compounds **2**, **4**, **5** and **7**) and stabilize the protein upon binding to it (compounds **1**, **2** and **4**). In both, the EMSA and FTS assays the influence exerted by the pathway unrelated salicylic acid (**4**) on LysRNt stability and LysRNt binding to DNA was the strongest among all tested compounds. Furthermore, LysRNt shares with other LTTRs amino acids putatively involved for binding of aromatic ligands.

### 3.4 LysRNt negatively influences brasilicardin biosynthesis in heterologous host *Amycolatopsis japonicum*

Due to the limited possibility to conduct genetic manipulations in native strain, we exploited the heterologous brasilicardin producer *Amycolatopsis japonicum* to examine the direct effect of LysRNt on the production of brasilicardin congeners. To obtain *lysRNt* overexpressing strain (*A. japonicum*::pPS1+pIJ_lysR), we cloned *lysRNt* into the integrative vector pIJ10257 under the control of the constitutive ermE* promoter (ermE*p) and subsequently introduced the resulting construct (pIJ10257_lysRNt) into a heterologous brasilicardin producer strain harboring the pPS1 fosmid (*A. japonicum*::pPS1) [8]. The pPS1 derivative of the original fosmid bcaAB01 confers biosynthesis of brasilicardin congeners; however, it misses all the putative regulatory genes (*lysRNt*, *sdpR*, *kstR*, *ompR*; Table S3) except *bra12*, encoding essential transcriptional activators of the Bra‐BGC. Total production of brasilicardin congeners (BraC, BraD, BraC‐agl and BraD‐agl) was measured, as described previously [8].

**FIGURE 4:**
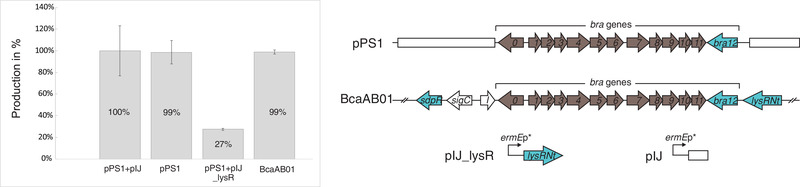
The influence of LysRNt on brasilicardins production. Total production of brasilicardin congeners (BraC, BraC‐agl, BraD, BraD‐agl) in *A. japonicum* strains harboring the constructs schematically depicted in the right panel. The average values of three technical replicates obtained for each strain were normalized to *A. japonicum*::pPS1+pIJ (100%), standard deviations were calculated using Excel spreadsheet and are presented on the graph

First, to exclude the possibility that the integration vector affects the brasilicardin production, we compared brasilicardin levels in the producer strains harboring the pPS1 fosmid with and without pIJ10257 integration vector (*A. japonicum*::pPS1+pIJ vs *A. japonicum*::pPS1). As demonstrated, the production levels were not altered in the presence of the integrative plasmid (Figure 4). Finally, to assess the effect of LysRNt, we analyzed brasilicardins production in the overexpression strain harboring *lysRNt* controlled by ermE*p (*A. japonicum*::pPS1+pIJ_lysR). The measurements revealed that the overexpression strain exhibited only ∼27% production level of the control strain (*A. japonicum*::pPS1+pIJ) containing an empty integrative vector, indicating that LysRNt exerts significantly a negative effect on brasilicardin production. Since, as shown above, the LysRNt binding sites are present within number of promoters of genes playing essential roles in brasilicardin biosynthesis (*bra0*, *bra1*, *bra7* and *bra12*). This indicates that LysRNt might negatively regulate the expression of these genes and thus hinder the biosynthesis of corresponding secondary metabolites. Interestingly, the presence of the *lysRNt* gene did not affect brasilicardin production in the strain harboring the full length fosmid bcaAB01, in comparison to the strain, which carries a pPS1 version (*A. japonicum*::bcaAB01 vs *A. japonicum*::pPS1) (Figure 4) (also reported in [8]) (see Section 4).

## 4 DISCUSSION

The recent use of heterologous host systems and genetic manipulations of the regulatory genes and precursor pathways turned out to be efficient in the improvement of production yield of brasilicardin congeners [8, 56], serving as a starting point for a semi‐synthetic approach. The latest advances in the total synthesis of brasilicardins (however, limited by low total yield) [57, 58] combined with a biosynthetic approach could allow further improvement of the production yields. However, the need for the improvement of brasilicardin congeners production yield in biological systems still seems to be desirable.

In this study, we identified LysRNt, a novel member of LysR‐type transcriptional regulators family being involved in the regulation of BraA biosynthetic pathway. Using EMSA, we have demonstrated that LysRNt interacts with promoters of the genes encoding enzymes involved in brasilicardin biosynthesis (*bra0*, *bra1* and *bra7*) and the essential TR (*bra12*) of the Bra‐BGC. These results suggested that LysRNt may either directly (via biosynthetic genes) or indirectly (via *bra12*), or both, regulate gene expression of the corresponding gene cluster and thus control brasilicardin biosynthesis. Assuming that Bra‐BGC comprises sub‐operons of putatively co‐transcribed genes (*bra1*‐*bra6* and *bra7*‐*bra11*), LysRNt binding to intergenic *bra0‐1* and *bra7* upstream regions could control expression of downstream genes involved in two distinct steps during brasilicardin biosynthesis. However, as only several promoters have been examined in this study, we cannot exclude LysRNt binding to other gene promoters within or near the Bra‐BGC. Interestingly, LysRNt does not bind, as many other LTTRs, to its own gene promoter indicating that it is not able to autoregulate its own expression.

In silico analyses allowed the prediction of two types putative LysRNt binding motifs (M1 and M2) within the bound promoter regions. Further experiments revealed that LysRNt specifically interacted with the M1 site lacking typical for LTTRs dyad sequence symmetry. However, it should be noted that several studies have already demonstrated that LysR‐type regulators recognize DNA sequences that differ significantly both in length and sequence or lack an obvious binding motif indicating that the classic binding box for LTTRs does not exist [32, 33]. The established using common bioinformatic tools sequence motif and logo probably need some further experimental verification as usually a larger set of input sequences is used to define a sequence logo. This information would allow more precise determination of exact number and organization of LysRNt binding sites within examined DNA fragments and could probably help explain different binding affinities of LysRNt to promoter regions observed in EMSA experiments. In many cases, the promoter regions bound by LTTRs contain a few binding sites of different affinity occupied by the regulators often in the signal dependent manner and contributing to transcription regulation [59]. It is very likely that additional possibly relaxed binding sequences, which were not identified in this study, may contribute to the overall LysRNt interaction with its target regions and may facilitate the formation of higher molecular nucleoprotein complexes. This idea could be supported by EMSA experiment demonstrating the preferential disassembly of the main nucleoprotein complex in the presence of the small‐compounds (e.g. samples A and A' in Figure 3A). We assume, this could indicate a protein loosening from its relaxed, low affinity binding sites, which suggests the interaction of LysRNt with additional binding sites within the studied DNA fragments. Similar effect, the release of the LTTR from its low affinity binding sites in the presence of the ligand have been described for CcpC protein and its ligand citrate [26]. LTTRs usually bind to multiple sites within target promoter regions, which is facilitated by regulator oligomerization. However, in our assays LysRNtHis_6_ existed only as monomer. Possibly some of LTTRs can also exist as monomers [60, 61]. However, we cannot also exclude the formation of LysRNt dimers or oligomers in different than used in our study experimental conditions (e.g. in the presence of DNA containing regulator binding sites or ligands). One of the explanations for LysRNt preference to exist as a monomer could be the relatively low conservation of the key amino acid residues within the presumable dimerization region between LysRNt regulator and other LTTRs (Figure 2C). Dimerization could also be hindered due to the presence of proximal His‐tag; however, it was demonstrated that C‐terminal His‐tags were unlikely to interfere with LTTRs’ activity, including protein‐protein interactions, in BenM and CatM [15, 17, 62, 63].

Using in vitro EMSA and FTS techniques, we have demonstrated that DNA‐binding activity of LysRNt can be negatively regulated by the compounds related with brasilicardin biosynthetic pathway (or being analogs of these compounds) and that some of these compounds can be possibly bound by LysRNt (e.g. salicylate or 3‐HBA). Interestingly, none of these compounds positively influenced DNA binding by the LysRNt, which is most common for LTTRs [24]. However, the opposite effect exerted by ligands has also been reported for this class of regulators, as in the mention above example of CcpC [26].

As demonstrated by FTS experiments, the highest stabilization effect on LysRntHis_6_ was exerted by the biosynthetic pathway‐unrelated compound salicylate (compound **4**), rather than the pathway‐related substrate compound 3‐HBA (compound **5**). Relaxed specificity for aromatic ligands has been previously observed for DntR regulator [64, 65, 66]. The DntR does not respond to the pathway substrate (4‐nitrobenzoate) but to the pathway‐unrelated salicylate, which induces DntR mediated transcriptional activation of cognate 4‐nitrobenzoate degradation pathway. For DntR, it was suggested that its specificity towards salicylate could be an evolutionary remnant of a common ancestor. Thus, the TR of biosynthetic/biodegradation pathway occurs along with the enzymes that have evolved a new substrate specificity. Alternatively, since salicylate and 3‐HBA are both the possible metabolites of chorismate pathway [67, 68], the salicylate could regulate brasilicardin biosynthetic pathway. LysRNt could also be involved in other metabolite pathways, for example those utilizing salicylate for biosynthesis of siderophores the potential virulence factors facilitating putatively *Nocardia farcinica* survival within a host [69].

Our studies indicate that in vitro interaction of LysRNt with ligands can alter its DNA‐binding activity and thus presumably affect transcription of controlled genes. Based on the fermentation studies, we assume that LysRNt negatively regulates gene expression within the Bra‐BGC which was reflected by the lower titer of brasilicardins in the *lysRNt* overexpression strain. However, exact role of this regulator has not been determined yet. Surprisingly, the *lysRNt* did not affect brasilicardins production when present on the full‐length fosmid (*A. japonicum*::bcaAB01) as compared to the strain lacking the *lysRNt* gene in the shortened version of the fosmid (*A. japonicum*::pPS1). However, it should be noted that the bcaAB01 fosmid harbors the full set of regulatory genes suggesting that other TRs (e.g. Bra12, KstR, OmpR or SdpR) could interfere with LysRNt regulatory function. They could act in one of the following ways: outcompete LysRNt from binding sites within promoters of biosynthetic genes or *bra12*; repress *lysRNt* gene expression; or both. However, experiments allowing determination of LysRNt regulator role in the production of brasilicardins in the native producer *N. terpenica* IFM 0406, including construction of *lysRNt* deletion mutant, are not yet possible due to lack of genetic tools. We assume that gene expression regulation in the Bra‐BGC is presumably a complex process that requires multiple regulators. Our results suggest, that this regulatory circuit includes also an impact exerted by the metabolites related with the brasilicardin biosynthetic pathway that can influence DNA binding activity at least of LysRNt transcriptional regulator and possibly other regulators. Considering the above aspects, we speculate that LysRNt could act as a switch that releases and de‐represses bound promoters in the presence of the metabolites (ligands) of the corresponding biosynthetic pathway to allow expression of the entire gene cluster and biosynthesis of the end‐product, BraA. Therefore, in the absence of these metabolites LysRNt could prevent firing the whole biosynthetic machinery at least until the specific inducing compounds reach a sufficient intracellular concentration. Transcriptional regulation of BGCs mediated via end‐products or intermediates has been described in several reports [39, 70–73]. Further in vivo studies are required to shed light on the possible role of small compounds in the regulation of LysRNt activity and brasilicardin biosynthesis.

## FUNDING

This study was funded by the National Centre for Research and Development (NCBR), Poland (grant no. ERA‐NET‐IB/NeBrasCa/10/2015) (Jolanta Zakrzewska‐Czerwińska); Ministerio de Economía y Competitividad (grant no. MINECO) (PCIN‐2014‐066) (Carmen Mendez); Ministerio de Economía y Competitividad (MINECO) (grant no. PCIN‐2014‐097) (Francisco Morris); Bundesministerium für Bildung und Forschung (BMBF) (grant no. FKZ 031A568A) (Harald Gross and Pierre Koch); Bundesministerium für Bildung und Forschung (BMBF) (grant no. FKZ 031A568B) (Wolfgang Wohlleben). The cost of publication was financed by the University of Wrocław, Faculty of Biotechnology statutory funds.

## ETHICAL STATEMENT

This article does not contain any studies with human participants or animals performed by any of the authors.

## CONFLICT OF INTEREST

The authors declare no conflict of interest.

## Supporting information

Supporting information
