# Supplementary material for: A novel LysR‐type regulator negatively affects biosynthesis of the immunosuppressant brasilicardin
Source: Eng Life Sci. 2020 Nov 4;21(1-2):4–18. doi: 10.1002/elsc.202000038 (PMC7837296; doi:10.1002/elsc.202000038)
Supplement: Supplementary file 1 — Supporting information [file ELSC-21-4-s001.pdf]

Engineering in Life Sciences

Supporting Information

A novel LysR-type regulator negatively affects biosynthesis of the immunosuppressant brasilicardin

Marcin Wolański[1*,#], Michał Krawiec[1,#], Paul N Schwarz[2], Evi Stegmann[2,3], Wolfgang Wohlleben[2,3], Anina Buchmann[3,4], Harald Gross[3,4], Michael Eitel[5], Pierre Koch[5], Alma Botas[6], Carmen Méndez[6], Luz Elena Núñez[7], Francisco Morís[7], Jesus Cortés[7] and Jolanta Zakrzewska-Czerwińska[1]

[#] these authors contributed equally to this work

* corresponding Author: Faculty of Biotechnology, University of Wrocław, ul. Joliot-Curie 14A, 50-383, Poland, e-mail: marcin.wolanski@uwr.edu.pl; tel.: +48 71 3752640

[1]*Faculty of Biotechnology, University of Wrocław, Wrocław, Poland;*
[2]*Department of Microbiology and Biotechnology, Interfaculty Institute of Microbiology and Infection Medicine, University of Tübingen, Tübingen, Germany*;
[3]*German Centre for Infection Research (DZIF), Partner Site Tübingen, Tübingen, Germany*;
[4]*Department of Pharmaceutical Biology, Institute of Pharmaceutical Sciences, University of Tübingen, Tübingen, Germany*;
[5]*Department of Pharmaceutical Chemistry, Institute of Pharmaceutical Sciences, University of Tübingen, Tübingen, Germany*;
[6]*Departamento de Biología Funcional e Instituto Universitario de Oncología del Principado de Asturias, Universidad de Oviedo, Oviedo, Spain*;
[7]*EntreChem S.L., Oviedo, Spain*

The list of supplementary materials

Supplementary figures

Fig. S1. LysRNt homologues.
Fig. S2. LysRNt dimerization interface.
Fig. S3. Purification of recombinant LysRNtHis$_6$ protein and oligomerization assay.
Fig. S4. Purification of recombinant LysRlnNtHis$_6$ protein.
Fig. S5. Putative sub-operon organization of biosynthesis genes within Bra-BGC and identification of LysRNt binding sites within Bra-BGC.
Fig. S6. Structures of small-compounds.
Fig. S7. Regulation of DNA binding activity and FTS assay.

Supplementary tables

Table S1. Plasmids and strains
Table S2. Oligonucleotides
Table S3. The list of total genes assigned on bcaAB01 fosmid and their putative functions
Table S4. Bioinformatic tools

Supplementary Materials and Methods

Supplementary Sequences

Supplementary References

Supplementary figures

Fig. S1. LysRNt homologues.
Result of BLASTp search against NCBI database performed using the LysRNt amino acid sequence. The top 100 hits, proteins sharing the highest sequence similarity with LysRNt are shown. The graph was generated using UGENE software based on the alignment result file exported from NCBI. The reference numbers (shown in the first column) are assigned to each record in the alignment; "Query_223016" refers to LysRNt amino acid sequence. The consensus sequence (ClustalW type) and the sequence conservation (grey bars) are shown on the top of the alignment.

Fig. S2. LysRNt dimerization interface.
A putative dimerization interface has been mapped to the LysRNt sequence using NCBI amino acid sequence alignment. The amino acid residues forming putative dimerization interface are marked with # and highlighted in yellow.

Fig. S3. Purification of recombinant LysRNtHis$_6$ protein.
(A) Affinity chromatography purification of LysRNtHis$_6$ protein. The protein was purified using HiTrap Talon® crude column (1 ml) and Äkta start system. The fractions collected during elution with increasing gradient of buffer B (2-50%) are indicated as F2 to F32. **(B)** SDS-PAGE analysis of LysRNtHis$_6$ fractions. The arrows indicate bands representing the corresponding LysRNtHis$_6$ protein (32.6 kDa). M – protein weight marker (26610, ThermoFisher Scientific). **(C)** Western Blot analysis of LysRNtHis$_6$ oligomerization assay results. The recombinant protein (~35 ng) was subjected to crosslinking in the presence or absence of BSA in reaction mixture (+ / -, respectively) for 5 and 20 minutes; the control samples "C" did not contain crosslinking agent. The samples were transferred onto membrane and visualized using anti-His antibody conjugated with horseradish peroxidase (SC-8036 HRP, Santa Cruz Biotechnology). The images were recorded using chemiluminescent (Chemi) and EPI white (EPI) modes. Positions of selected marker bands are indicated using dotted lines. M – prestained protein weight marker (26616, ThermoFisher Scientific).

Fig. S4. Purification of recombinant LysRlnNtHis$_6$ protein.
(A) Affinity chromatography purification of LysRNtlnHis$_6$ protein. The protein was purified using HiTrap Talon® crude column (1 ml) and Äkta start system. The fractions collected during elution with increasing gradient of buffer B (2-50%) are indicated as F2 to F20. **(B)** SDS-PAGE analysis of LysRlnNtHis$_6$ fractions. The arrows indicate bands representing the corresponding LysRlnNtHis$_6$ protein (35.4 kDa). M – protein weight marker (26610, ThermoFisher Scientific). **(C)** Western Blot analysis of LysRlnNtHis$_6$. The protein transferred onto membrane was visualized using anti-His antibody conjugated with horseradish peroxidase (SC-8036 HRP, Santa Cruz Biotechnology). The images were recorded using chemiluminescent (Chemi) and EPI white (EPI) modes. Positions of selected marker bands on Chemi image are indicated using dotted lines. M – protein weight marker (26616, ThermoFisher Scientific).

Fig. S5. Putative sub-operon organization of biosynthesis genes within Bra-BGC and identification of LysRNt binding sites within Bra-BGC.
(A) Graphical depiction of the brasilicardin biosynthetic gene cluster illustrating the presumable sub-operon organization of Bra-BGC and intergenic 179-bps gap between *bra6* and *bra7*. The numbers in green and red indicate the number of gap forming or overlapping nucleotides, respectively. **(B)** *In silico* determination of LysRNt binding sequences using MEME. The putative M1 and M2 LysRNt binding sites are shown with light violet (M1) and light red (M2) boxes. The general and the detailed

pictures are shown. The lengths of given DNA probes (black solid lines) and intergenic regions (red solid lines) are shown. **(C)** Site specific mutagenesis of M1 and M2 sites.

Fig. S6. Structures of small-compounds.

(A) Chemical structures of brasilicardin A (BraA), brasilicardin B (BraB), brasilicardin C aglycone (BraC-agl), brasilicardin D aglycone (BraD-agl), brasilicardin C (BraC), brasilicardin D (BraD) (based on Schwarz et al. [1]). **(B)** Biosynthetic pathway of brasilicardin A (based on Schwarz et al. [1]). Single and multiple biosynthetic steps are marked with solid and dotted line arrows, respectively. Substrates and products of biosynthetic pathway are given in frames, enzymes catalyzing reactions are given along the arrows. **(C)** Chemical structures of ligands bound by some LysR-type transcriptional regulators (LTTRs).

Fig. S7. Regulation of DNA binding activity and FTS assay.

(A) Cross-linked assay. EMSA showing interaction of LysRNtHis$_6$ protein (500 nM) with bra12p DNA fragment in the presence of compounds 1, 2, 4, 5, A and C (1 = methyl 4-hydroxybenzoate, 2 = sodium benzoate, 4 = salicylic acid, 5 = 3-hydroxybenzoic acid, 6 = BraC-aglycone, 7 = BraC). Two different concentrations, 1 and 2 mM, of compounds were used; samples containing 2 mM concentration of given compounds have been marked with an apostrophe. All the reaction mixtures contained: DNA, LysRNtHis$_6$ protein and either a given compound diluted in EtOH (final concentration 1%) or, in the case of control sample (Et), the EtOH (1%). The (-) reactions contained EtOH but lacked LysRNt protein. Samples shown were cross-linked using glutaraldehyde (0.5 mM) before electrophoresis. The free DNA and the DNA in complex with protein were marked by short solid black lines without and with black circle on it, respectively. The numbers shown below each gel indicate intensities of DNA in protein-DNA complexes expressed as a percentage of protein-DNA complex measured in corresponding control lanes (Et). The areas used for calculations are limited by dotted lines. The reference lanes (100%) are highlighted with black background. The samples containing 3-HBA analogs has been highlighted with grey background in this and subsequent panels. **(B)** Intensities of DNA bound to LysRNtHis$_6$ presented in panel A. **(C)** The FTS (fluorescence thermal shift) demonstrating recombinant LysRNtHis$_6$ at 2 µM concentration without supplementation with small-compounds. The plot represents three technical replicates.

Supplementary tables

Table S1. Plasmids and strains

**Plasmids**		
Name	Description	Source / Reference
pET-21a(+)	Expression vector	Laboratory stock / (Merck)
pET-21a(+)lysRNt	Expression vector pET-21a(+) derivative carrying *lysRNt* gene	This study
pET-21a(+)lysRlnNt	Expression vector pET-21a(+) derivative carrying *lysRlnNt* gene	This study
pIJ10257	Integrative *Streptomyces* expression vector based on the strong, constitutive *ermE* promoter (p*ermE*), HygR	JIC Norwich UK / [2]
pIJ10257_lysRNt	Integrative vector pIJ10257 derivative carrying *lysRNt* gene under control of p*ermE*	This study
bcaAB01 fosmid	pCC1FOS (Epicentre): Cam^R resistance exchanged with Kam^R, vector containing the brasilicardin biosynthetic gene cluster	[1]
pPS1	Truncated bcaAB01 derivative, carrying only the brasilicardin gene cluster and the bra12 gene	[1]
pTZ57R/T	Cloning vector	Laboratory stock / (ThermoFisher Scientific)
pTZ/5_3 (delta_M1_bca01p)	Cloning vector pTZ57R/T derivative carrying ΔM1_bra0-1p fragment	This study
pTZ/8_7 (delta_M2_bca01p)	Cloning vector pTZ57R/T derivative carrying ΔM2_bra0-1p fragment	This study
pTZ/bra8p (1)	Cloning vector pTZ57R/T derivative carrying bra8p fragment	This study
pTZ/1WT (WT_bca01p)	Cloning vector pTZ57R/T derivative carrying wild-type bra0-1p fragment	This study
**Strains**		
Name	Description	Source / Reference
Escherichia coli ET12567/pUZ8002	*E. coli: dam*, *dcm*, *hsdS*, Cam^R, Tet^R containing plasmid pUZ8002: *tra*, Kan^R, *RP4 23*	[3, 4]
E. coli BW25113/pIJ790	Δ(*araD-araB*)*567* Δ*lacZ4787*(::*rrnB4*) *lacIp-40000*(*lacI*^{q}) λ$^-$ *rpoS369*(Am) *rph-1* Δ(*rhaD rhaB*)*568 hsdR514* on the bacterial chromosome; *oriR101 repA1001*(Ts) *araBp-gam-be-exo* on the pIJ790 plasmid	[5]
E. coli Rosetta^{TM} 2(DE3)	F$^-$ *ompT hsdS*$_B$(r$_B^-$ m$_B^-$) *gal dcm* (DE3) pRARE2 (Cam^R)	Laboratory stock / (Merck)
Amycolatopsis japonicum MG417-CF17	Wild type	[6]
A. japonicum::bcaAB01	Wild type *A. japonicum* with chromosomally integrated bcaAB01 fosmid	[1]
A. japonicum::pPS1	Wild type *A. japonicum* with chromosomally integrated pPS1 fosmid	[1]
A. japonicum::pPS1+pIJ	Wild type *A. japonicum* with chromosomally integrated pPS1 fosmid and pIJ10257 empty vector	This study
A. japonicum::pPS1+pIJ_lysR	Wild type *A. japonicum* with chromosomally integrated pPS1 fosmid and pIJ10257_lysRNt vector	This study

Table S2. Oligonucleotides

Name	Sequence (5’>3’)	Application
Cloning		
FP_lysRpET21_2	CTTTAAGAAGGAGATATACATATGGAGTGGTCCTCGACCG	Cloning of *lysRNt* gene into pET-21a(+) vector. NdeI site is underlined
RP_lysRpET21	AGTGGTGGTGGTGGTGGTGCGGCAGCGGCAG	Cloning of *lysRNt and lysRlnNt* genes into pET-21a(+) vector
FP_lysRpET21	CTTTAAGAAGGAGATATACATATGGGCCGCTCCG	Cloning of *lysRlnNt* gene into pET-21a(+) vector. NdeI site is underlined
lysRbis_pIJ_fw	GTCTAGAACAGGAGGCCCCATATGGAGTGGTCCTCGACCG	Cloning of *lysRNt* gene into pIJ10257 vector. NdeI site is underlined
lysRbis_pIJ_rev	GACTCTAGTTAATTAATCACTCACGGCAGCGGCAGG	Cloning of *lysRNt* gene into pIJ10257 vector.
Other purposes		
sdpRp_1rv	CGTCGAACGCTACTGTCACAACCGC	EMSA, amplification of sdpRp fragment
sdpR_fw1	GTCGACTCCATCCGCTCCGCG	EMSA, amplification of sdpRp fragment
bca1-0p_fw1	GGTCGCAGGCATCGATGACCCC	EMSA, amplification of bra0-1p fragment
bca1-0p_rv1	CCGCCGCCCTCGCCAC	EMSA, amplification of bra0-1p fragment
bca7p_fw1	ACCAGTGCCTCGGCCAGGG	EMSA, amplification of bra7p fragment
bca7p_rv1	ACCCGCTGCTCAATCCCACCAC	EMSA, amplification of bra7p fragment
bca8p_fw1	GTGGAGGTCGAGACGGTGCTGT	EMSA, amplification of bra8p fragment
bca8p_rv1	CGCGCCGCCCGGTCAC	EMSA, amplification of bra8p fragment
afsRp_rv1	AGCCCCAGCCGAACCAGTGACAT	EMSA, amplification of bra12p fragment
afsRp_fw1	GGTCGCCGCCGGGTACG	EMSA, amplification of bra12p fragment
lysRp_1rv	CGGTCGAGGACCACTCCAT	EMSA, amplification of lysRNtp fragment
lysRp_1fw	CGGCTGTCGCTGACCGAA	EMSA, amplification of lysRNtp fragment
del_M1_bca01_up	TAATCCTCGAGCGGCATCGAAACCGGTCATATTG	Mutagenesis of M1 LysRNt site within bra0-1p fragment
del_M1_bca01_dn	CGATGCCGCTCGAGGATTATTGTCCGGAAGCATTGT	Mutagenesis of M1 LysRNt site within bra0-1p fragment
del_M2_bca01_up	GGTGAGTTTCCTCTAGAATATCACGGCGAAACCGG	Mutagenesis of M2 LysRNt site within bra0-1p fragment
del_M2_bca01_dn	GTGATATTCTAGAGGAAACTCACCAATTCATGATCG	Mutagenesis of M2 LysRNt site within bra0-1p fragment
pTZCy5	TCGGTACCTCGCGAATGCATC	Cy5 labeled fluorescent primer, amplification of fluorescently labeled fragments cloned into pTZ57R/T vector

Table S3. The list of total genes assigned on bcaAB01 fosmid and their putative functions.

Genes are listed in the reversed order of appearance in the genome. Gene annotations (locus tags) are available in the NCBI Genome Database under LWGR00000000.1 GenBank accession number. To get direct access to nucleotide and protein sequences use the following link: [*https://www.ncbi.nlm.nih.gov/genome/proteins/14487?genome_assembly_id=274145*](https://www.ncbi.nlm.nih.gov/genome/proteins/14487?genome_assembly_id=274145)

Locus tag no.	Gene name	Function	Protein product no.	Protein function description
AWN90_RS33480	*kstR*	R	WP_067591063.1	TetR/AcrR family transcriptional regulator, AcrR-like
AWN90_RS33475	-	O	WP_067591060.1	Putative agmatine deiminase
AWN90_RS33470	-	O	WP_067591058.1	N-carbamoylputrescine amidase
AWN90_RS33465	-	O	WP_082871583.1	purine/cytosine permease
AWN90_RS33460	-	O	WP_067591055.1	amidohydrolase
AWN90_RS33455	-	O	WP_067591052.1	mycolyltransferase
AWN90_RS33450	-	O	WP_067591049.1	NADP-dependent oxidoreductase
AWN90_RS33445	-	O	WP_082871892.1	diguanylate cyclase
AWN90_RS33440	-	U	WP_067591046.1	hypothetical protein
AWN90_RS33435	-	U	WP_067591043.1	hypothetical protein
AWN90_RS33430	-	U	WP_067591040.1	hypothetical protein
AWN90_RS33425	-	U	WP_067591037.1	hypothetical protein
AWN90_RS33420	*sdpR*	R	WP_067591034.1	SdpR/ArsR family transcriptional regulator
AWN90_RS33415	*sigC*	O	WP_082871582.1	RNA polymerase σ-70 factor, ECF subfamily
AWN90_RS33410	*/*	U	WP_082871891.1	hypothetical protein (integral membrane protein)
AWN90_RS33405	*bra0*	B	WP_067591028.1	dioxygenase
AWN90_RS33400	*bra1*	B	WP_082871581.1	Putative phenylalanine aminotransferase
AWN90_RS33395	*bra2*	B	WP_082871580.1	Geranylgeranyl diphosphate synthase
AWN90_RS33390	*bra3*	B	WP_067596046.1	prenyltransferase
AWN90_RS33385	*bra4*	B	WP_067591021.1	Diterpene cyclase
AWN90_RS33380	*bra5*	B	WP_067591017.1	Putative epoxidase
AWN90_RS33375	*bra6*	B	WP_082871579.1	P450
AWN90_RS33370	*bra7*	B	WP_067591014.1	Putative 2,3-dihydroxybenzoate-AMP ligase
AWN90_RS33365	*bra8*	B	WP_082871578.1	Putative oxidase
AWN90_RS33360	*bra9*	B	WP_067591011.1	Hypothetical protein (acyltransferase)
AWN90_RS33355	*bra10*	B	WP_067591007.1	Putative rhamnosyltransferase
AWN90_RS33350	*bra11*	B	WP_067596043.1	O-methyltransferase
AWN90_RS33345	*bra12*	R	WP_082871577.1	SARP-like transcriptional regulator
AWN90_RS33340	*lysRNt*	R	WP_067591001.1	Putative LysR-like transcriptional regulator
AWN90_RS33335	-	O	WP_067590998.1	isocitrate lyase/carboxyvinyl-carboxyphosphonate phosphorylmutase
AWN90_RS33330	*ompR*	R	WP_067590995.1	response regulator transcription factor
AWN90_RS33325	-	O	WP_082871576.1	sensor-type histidine kinase
AWN90_RS33320	-	U	WP_067590992.1	hypothetical protein
AWN90_RS33315	-	O	WP_067590989.1	FAD-dependent monooxygenase

Function:
R – regulatory
B – brasilicardin biosynthesis
U – unknown
O – other functions

Table S4. Bioinformatic tools

Software name	Web address	References
SMART	http://smart.embl-heidelberg.de/	[7, 8]
PSIPRED v3.3	http://bioinf.cs.ucl.ac.uk/psipred/	[9, 10]
ProtParam	https://web.expasy.org/protparam/	[11]
UGENE	http://ugene.net/	[12]
blastp	https://www.ncbi.nlm.nih.gov/	
Phyre 2.0	http://www.sbg.bio.ic.ac.uk/phyre2/	[13]
MEME	http://meme-suite.org/tools/meme	[14]
WebLogo	https://weblogo.berkeley.edu/	[15]
Pattern Locator	https://www.cmbl.uga.edu//software/patloc.html	[16]
UniProtKB	https://www.uniprot.org/help/uniprotkb	[17]

Supplementary Materials and Methods

Vector and strain construction (detailed)

Construction of pET-21a(+)lysRNt

The *lysRNt* gene was cloned in pET-21a(+) vector to obtain pET-21a(+)lysRNt using SLIC. To amplify the *lysRNt* gene the PCR reaction was conducted with "FP_lysRpET21_2" and "RP_lysRpET21" primers (Table S2) using *Nocardia terpenica* IFM 0406 strain chromosomal DNA as template. The PCR product (insert) was subsequently purified from a gel and cloned into the pET-21a(+) digested with NdeI and XhoI (vector) restriction enzymes using SLIC [18]. The SLIC reaction was conducted using T4 DNA polymerase as follows: 0.06 pmols of purified PCR product (insert), 0.03 pmols of linearized plasmid DNA (vector) and 1 U of T4 DNA polymerase (EP0061, ThermoFisher Scientific) were mixed in T4 DNA polymerase buffer and incubated for 5 min at 25°C in the final volume of 10 µl; the reactions were stopped by addition of dCTP (1 µl, 10 mM) and incubated for 5 min at 25°C; the reaction mixtures were used directly for transformation of chemically competent *E. coli* DH5α cells using standard protocol. Plasmids isolated from single clones were verified by restriction digestion and DNA sequencing.

Construction of pET-21a(+)lyslnRNt (long version)

The extended version of *lysRNt* gene (*lysRlnNt*) was cloned into pET-21a(+) vector to obtain pET-21a(+)lysRlnNt. To amplify the corresponding gene using PCR the following primers were used: "FP_lysRpET21" and "RP_lysRpET21" (Table S2). The cloning procedure was the same as described above for pET-21a(+)lysRNt. The clones were verified using DNA sequencing.

Construction of pIJ10257_lysRNt vector

The *lysRNt* gene was cloned into pIJ10257 vector to obtain pIJ10257_lysRNt using SLIC. The *lysRNt* gene was PCR amplified with "lysRbis_pIJ_fw" and "lysRbis_pIJ_rev" primers (Table S2) and *Nocardia terpenica* IFM 0406 chromosomal DNA. The PCR product was purified from a gel and cloned into the pIJ01257 vector digested with NdeI and XhoI restriction enzymes using SLIC reaction as described above. Resulting clones were verified by restriction analysis and DNA sequencing.

Mutagenesis of M1 and M2 sites

The ∆M1_bra0-1p and ∆M2_bra0-1p were generated using overlap extension PCR with the following combinations of primers, bca1-0p_fw1 / del_M1_bca01_dn and del_M1_bca01_up / bca1-0p_rv1, to generate ∆M1_bra0-1p fragment; bca1-0p_fw1 / del_M2_bca01_dn and del_M2_bca01_up / bca1-0p_rv1, to generate ∆M2_bra0-1p fragment. The PCR products were subsequently cloned into pTZ57R/T vector and verified by sequencing.

Protein purification (detailed)

Protein LysRNtHis$_6$ purification

The recombinant LysRNtHis$_6$ protein was expressed in the *E. coli* Rosetta™ 2(DE3) strain (Merck) transformed with the pET-21a(+)lysRNt vector. To produce LysRNtHis$_6$ protein an overnight preculture (100 ml of TB medium supplemented with ampicillin (200 µg/ml) and chloramphenicol (34 µg/ml)) was prepared and subsequently transferred at 1/5 ratio to a fresh portion of TB medium supplemented with ampicillin (100 µg/ml). The cultures were conducted in baffled flasks (800 ml TB medium in 2.8-liter flask) in the incubator shaker (180 rpm) at the following scheme: incubation for 7 hours at 37°C; transfer to 20°C and supplementation with 5xTB concentrate (80 ml per 800 ml TB); protein production induction with IPTG (1 mM) (isopropyl-b-D-thio-215 galactoside); overnight incubation at 20°C. Cultures were then harvested and the cell pellet was stored at -20°C. For each

protein purification procedure 1.6-litre culture was prepared. Cells stored at -20°C cells were thawed on ice before use and resuspended in lysis buffer A (50 mM NaH_2PO_4, 300 mM NaCl, 10 mM imidazole, pH 8.0) (5 ml/g of cell paste) supplemented with protease inhibitors (A32965, ThermoFisher Scientific) and universal nuclease (88702, ThermoFisher Scientific), then the cells were disrupted by sonication (Sonics) (5s ON/ 5s OFF, amplitude 40-50%, 10 minutes sonication time) and clarified by centrifugation (45 min., 37 000 x g, 4°C). LysRNtHis$_6$ protein was purified by affinity chromatography using HiTrap Talon® crude column (1 ml, GE Healthcare) with Äkta start system (GE Healthcare) according to system built-in protocol with minor modifications. Recombinant protein was eluted from the resin using increasing gradient of elution buffer B (50 mM NaH_2PO_4, 300 mM NaCl, 500 mM imidazole, pH 8.0) in lysis buffer A. Protein fractions were examined using sodium dodecyl sulfate-polyacrylamide gel electrophoresis (SDS-PAGE), similar fractions were then pooled together and aliquoted. Protein samples were flash frozen with liquid nitrogen and stored at -80°C.

Protein LysRlnNtHis$_6$ (long version) purification

The expression and purification procedures were the same as described above for LysRNtHis$_6$ protein.

Western Blot

Following electrophoresis, the proteins were transferred onto the nitrocellulose blotting membrane (Amersham^{TM} Protran^{TM} 0.45 µm NC, GE Healthcare Life Science) using PierceG2 Fast Blotter mixed range mode (ThermoFisher Scientific). Upon the transfer membrane blocked for at least 1 h using powder skim milk dissolved (3% w/v) in TBST (10 mM Tris-HCl pH 8.0, 150 mM NaCl, 0.05% Tween 20) buffer. Following blocking, the membrane was incubated with anti-His mouse monoclonal antibody conjugated with horseradish peroxidase (SC-8036 HRP, Santa Cruz Biotechnology) according to manufacturer delivered protocol. The proteins were visualized using WesterBright^{TM} Sirius chemiluminescence detection kit (Advansta) according to manufacturer instructions. The images were recorded using ChemiDoc MP system (Bio-Rad).

Electrophoretic mobility shift assay (detailed)

EMSA protocol for ^{32}P radiolabeled DNA

The LysRNtHis$_6$ protein was incubated with 50 cps ^{32}Pradiolabeled DNA (^{32}P) (~ 1.5 fmol). The binding reactions were conducted in 20 µl in 1× phosphate-buffered saline (PBS; 137 mM NaCl, 2.7 mM KCl, 10 mM Na_2HPO_4, 1.8 mM KH_2PO_4) supplemented with bovine serum albumin (BSA) and glycerol (5 µg/µl and 10%, respectively) and non-specific competitor poly(dI-dC)·(dI-dC) (3.75 ng/µl) for 30 minutes at 25°C. Before sample application the gel was pre-run for 30 min at 100 V 4-8°C and ~3 h at the same conditions upon sample application. Upon completion of the electrophoresis, the gel was transferred to a single sheet of Whatmann 3MM paper, dried using vacuum gel dryer and incubated overnight with Storage Phosphor Screen (GE Healthcare). The screen was subsequently analyzed with autoradiography reader (Typhoon 8600 Variable Mode Imager) and Image Quant software (both, GE Healthcare). The gels used for that type of experiment were 15 cm long, 5% native polyacrylamide (37.5:1, 3029.2 Carl Roth) in 0.25x TBE.

To analyze the influence of substrates and semi-products of brasilicardin biosynthesis on LysRNt ability to interact with DNA the reactions were supplemented with 1 µl of respective compound (diluted in 20% EtOH) in a final volume of 20 µl. The small-compounds were added to reaction mixtures at the initial stage of incubation. Depending on the experimental approach the samples were directly run on a gel or crosslinked with glutaraldehyde before electrophoresis. In the second

option samples were crosslinked with glutaraldehyde solution (1 µl 10 mM added to 20 µl reaction) for 5 min at 25°C and transferred on ice.

EMSA protocol for fluorescently labeled DNA

The fluorescently labeled DNA fragments (bra0-1p, ΔM1_bra0-1p, ΔM2_bra0-1p and bra8p) were PCR amplified using pTZCy5 and respective reverse primer (bca1-0p_rv1 or bca8p_rv1) using Q5 polymerase and appropriate pTZ57R/T plasmid template. After completion the PCR mixtures were treated with exonuclease I (ExoI, ThermoFisher Scienitific) and thermosensitive alkaline phosphatase (FastAP, ThermoFisher Scienitific) (15 min, 37°C) and column purified (Clan-Up, A&A Biotechnology).

The LysRNtHis$_6$ protein was incubated with 20 ng fluorescently labeled DNA in the same reaction conditions as applied for radiolabeled DNA fragments. The only changes were different protein concentrations (indicated within the manuscript text) and higher concentration of non-specific competitor poly(dI-dC)·(dI-dC), 12.5 ng/µl . The gel was resolved as described above. Upon completion of electrophoresis the gel was placed directly onto the glass stage of the imaging system (Azure C600) and the image was recorded using default settings for chemiluminescent Western blots.

<u>Oligomerization assay</u>

Recombinant LysRNtHis$_6$ protein (100 ng) was incubated at room temperature (ca. 25°C) in 20 µl in 1× phosphate-buffered saline (PBS; 137 mM NaCl, 2.7 mM KCl, 10 mM Na_2HPO_4, 1.8 mM KH_2PO_4) supplemented with glycerol (10%), and with or without bovine serum albumin (BSA) (5 µg/µl) for 30 minutes, followed by addition of 1 µl of glutaraldehyde solution (10 mM in PBS buffer) and incubation for 5 and 20 min. The control samples were incubated for designated time without glutaraldehyde. Reactions were stopped at indicated time points by the addition of 1 µl of TRIS buffer (1M, pH 8.0) and the whole reaction mixture was transferred on ice. The samples were then stored at -20°C until the time of use. Subsequently, the samples were mixed with SDS-PAGE sample buffer and resolved on 10% polyacrylamide gels using standard sodium dodecyl sulfate-polyacrylamide gel electrophoresis (SDS-PAGE) procedure (Laemmli 1970). Following electrophoresis, the proteins were transferred onto the nitrocellulose blotting membrane and visualized using Western Blot protocol.

Supplementary Sequences

1. Sequence of native LysRNt protein

LysRNt (303 amino acids, 31.8 kDa, pI 5.54)

MEWSSTALRVLRAIAELGSFTAAAGALGYTQSAVSRQMAVLEHAAGTPLFERRSGGATLTAAGATLLRHASAALDDLDRAERAL
HGIEPGGGTVRVGVFTSIGAAVLPETLTLMRRRRPDVDVVTREGSTAALTRSLRAGTLDLAVISARPPYPAPDDQDPPLELDVLLE
GELLVAVPADSRLGRDGTVGLDDLQTATWVSGPHTTGEHGIGVWPALPQRPIIGHQTRDWLSKLSLVAAGYGVTTLPPYLLAL
VPDTVRVVRVVEGAPVTRRVLLARLPGLITDAVDDLAACLRDAIDGLPLP

2. Sequence of recombinant LysRNtHis$_6$ protein

LysRNtHis$_6$ (309 amino acids, 32.6 kDa, pI 6.04)

MEWSSTALRVLRAIAELGSFTAAAGALGYTQSAVSRQMAVLEHAAGTPLFERRSGGATLTAAGATLLRHASAALDDLDRAERAL
HGIEPGGGTVRVGVFTSIGAAVLPETLTLMRRRRPDVDVVTREGSTAALTRSLRAGTLDLAVISARPPYPAPDDQDPPLELDVLLE
GELLVAVPADSRLGRDGTVGLDDLQTATWVSGPHTTGEHGIGVWPALPQRPIIGHQTRDWLSKLSLVAAGYGVTTLPPYLLAL
VPDTVRVVRVVEGAPVTRRVLLARLPGLITDAVDDLAACLRDAIDGLPLPHHHHHH

3. Sequence of alternative LysRlnNt protein (long version)

LysRlnNt (328 amino acids, 34.6 kDa, pI 5.72)

MGRSGLFAINLLRNDGFDCRVCCFAMEWSSTALRVLRAIAELGSFTAAAGALGYTQSAVSRQMAVLEHAAGTPLFERRSGGAT
LTAAGATLLRHASAALDDLDRAERALHGIEPGGGTVRVGVFTSIGAAVLPETLTLMRRRRPDVDVVTREGSTAALTRSLRAGTLD
LAVISARPPYPAPDDQDPPLELDVLLEGELLVAVPADSRLGRDGTVGLDDLQTATWVSGPHTTGEHGIGVWPALPQRPIIGHQT
RDWLSKLSLVAAGYGVTTLPPYLLALVPDTVRVVRVVEGAPVTRRVLLARLPGLITDAVDDLAACLRDAIDGLPLP

The N-terminal 25 amino acid extension is underlined. This extension represents additional amino acid present in the putative LysRlnNt protein but not in the native LysRNt protein.

Supplementary References

[1] Schwarz PN, Buchmann A, Roller L, Kulik A, Gross H, Wohlleben W, Stegmann E. 2018. Biotechnol J 13:1–12.

[2] Hong H-J, Hutchings MI, Hill LM, Buttner MJ. 2005. J Biol Chem 280:13055–13061.

[3] Kallifidas D, Paget M. 2010.

[4] MacNeil DJ, Gewain KM, Ruby CL, Dezeny G, Gibbons PH, MacNeil T. 1992. Gene 111:61–68.

[5] Gust B, Challis GL, Fowler K, Kieser T, Chater KF. 2003. Proc Natl Acad Sci U S A 100:1541–6.

[6] Nishikiori T, Okuyama A, Naganawa H, Takita T, Hamada M, Takeuchi T, Aoyagi T, Umezawa H. 1984. J Antibiot (Tokyo) 37:426–427.

[7] Letunic I, Bork P. 2018. Nucleic Acids Res 46:D493–D496.

[8] Schultz J, Milpetz F, Bork P, Ponting CP. 1998. Proc Natl Acad Sci U S A 95:5857–5864.

[9] Buchan DWA, Jones DT. 2019. Nucleic Acids Res 47:W402–W407.

[10] Jones DT. 1999. J Mol Biol 292:195–202.

[11] Wilkins MR, Gasteiger E, Bairoch A, Sanchez JC, Williams KL, Appel RD, Hochstrasser DF. 1999. Methods Mol Biol.

[12] Okonechnikov K, Golosova O, Fursov M, Varlamov A, Vaskin Y, Efremov I, German Grehov OG, Kandrov D, Rasputin K, Syabro M, Tleukenov T. 2012. Bioinformatics.

[13] Kelley LA, Mezulis S, Yates CM, Wass MN, Sternberg MJE. 2015. Nat Protoc 10:845–858.

[14] Bailey TL, Boden M, Buske FA, Frith M, Grant CE, Clementi L, Ren J, Li WW, Noble WS. 2009. Nucleic Acids Res 37:W202–W208.

[15] Crooks GE, Hon G, Chandonia JM, Brenner SE. 2004. Genome Res 14:1188–1190.

[16] Mŕazek J, Xie S. 2006. Bioinformatics 22:3099–3100.

[17] Bateman A. 2019. Nucleic Acids Res 47:D506–D515.

[18] Li MZ, Elledge SJ. 2007. Nat Methods 4:251–256.

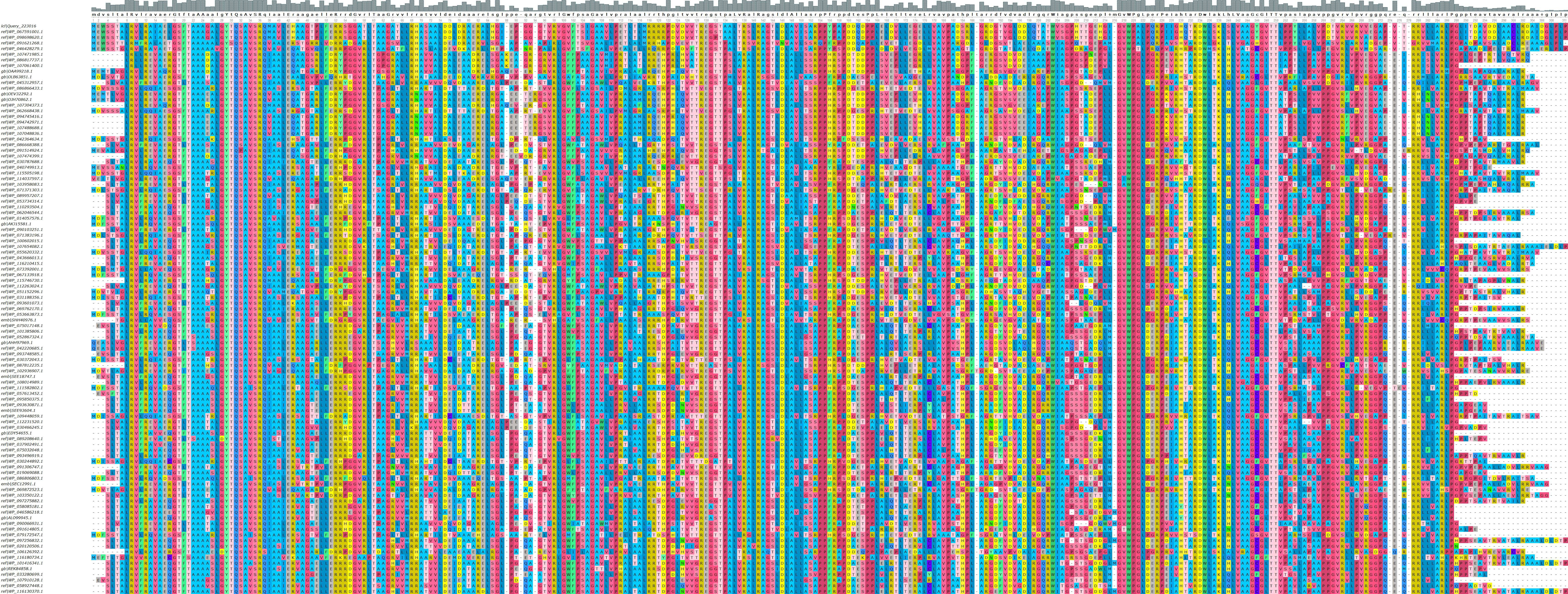

```
                                   ## ##  #   #     #######
gi 117165276  93 TVTVASF.[2].GIALVVAPALGRLAESAP.[1].IRIRVQD.[7].PMVLDR.[1].VDVAV.[14].LTHVPLYA 168
LysRNt        93 TVRVGVF.[2].IGAAVLPETLTLMRRRRP.[1].VDVVTRE.[7].RSLRAG.[1].LDLAV.[16].LELDVLLE 170
gi 150397298  92 ELRVAAF.[2].VAGAVVAGAIHGLHRLHP.[1].LTVQFDE.[7].AALRSW.[1].TDIAI.[14].IETIPLME 167
gi 111017595  94 RLTVGVW.[2].VASSLVPSALTLLAAEHP.[1].IEVRTRE.[7].VAVRDG.[1].LDFSF.[14].LTRVVIAV 169
gi 120609006  92 ELRVAAF.[2].IASALLPETIQALRAAYP.[1].LRLAVEE.[7].AALGSW.[1].ADIAL.[14].YGFVALAR 167
gi 152966772 102 HLSLAYI.[2].VGSTWLPEVARRLTQAHP.[1].VDLDLQL.[6].EGTERP.[1].VQLVV.[11].FDVHRLAE 173
gi 134096962  93 TIRICAF.[2].ASATIVPAATASATRAHP.[1].LRIRLVE.[7].ETLRRG.[1].CDIAL.[14].VIGIEVLE 168
gi 134102712  94 RLRLGAF.[2].AGLALLPDVLAALRHRHP.[1].AELSVVD.[7].ALVGSH.[1].LDLAL.[13].VRLAPLID 168
gi 226364736  93 MLAIGTF.[2].AGSSLLPLVVKEFKTHHP.[1].IDLRVLS.[7].ESLRRR.[1].TELSL.[13].LTCHHLMN 167
gi 119963461  92 RFGLGGF.[2].AAAQLLAPLAATLRSTRP.[1].LEVQVLE.[7].DLLVAE.[1].IDLAV.[15].FEQRVLLD 168

                                                           ######        ## ##
gi 117165276 169 .[1].PFDAVVPVAHR.[8].LAELAKDPWIGP.[20].PRMEHSSDDF.[2].VVSLAAAD.[1].GVALVPRS.[8]. 257
LysRNt       171 .[1].ELLVAVPADSR.[8].LDDLQTATWVSG.[18].PIIGHQTRDW.[2].KLSLVAAG.[1].GVTTLPPY.[7]. 256
gi 150397298 168 .[1].VFNVMVSRTHP.[8].LHQLRNERWALD.[20].PNIVARCKGF.[2].TIALIRQG.[1].GISILPGL.[7]. 255
gi 111017595 170 .[1].RLHAALPPGTV.[6].LADLAEHPWILA.[20].PRINHEVGEQ.[2].ALAMVAAG.[1].GVTLVSDL.[7]. 255
gi 120609006 168 .[1].SLHVLLPATHP.[8].VADLRDEPWAMD.[20].PRMDVRALGF.[2].VSAMVGAG.[1].AVTVVPGL.[7]. 255
gi 152966772 174 .[1].PYRVLLPATHP.[8].LAELATDRWIDS.[21].PAFHVQAHDY.[2].SAAFVGAG.[1].GITVLPAL.[7]. 262
gi 134096962 169 .[1].PVVAVLPTGHR.[8].LAELRGERWIAG.[18].PDIAFTTDDN.[2].VQSLVAAG.[1].GIALMPRL.[8]. 255
gi 134102712 169 .[1].PVHAVLPTDHR.[8].LADFADEPWAAA.[20].PNVPFESESY.[2].AEAVVSAG.[1].AVAFIPRL.[6]. 255
gi 226364736 168 .[1].PTLLLVSEKHR.[8].ITELRDESWVVR.[20].PRTSFLAHDY.[2].VQAMVGVE.[1].GVAIAPRL.[7]. 255
gi 119963461 169 .[1].PLDVIIPGDHP.[8].LEELASEPWITE.[20].PRIAHEAVEW.[2].QIAFVGAG.[1].GVGLLPRL.[7]. 256

gi 117165276 258 GVVVRPVD.[4].TRRVFAAVRR.[15]. 294
LysRNt       257 TVRVVRVV.[5].TRRVLLARLP.[15]. 294
gi 150397298 256 DVWVCKLE.[3].RRKISLAFRK.[15]. 291
gi 111017595 256 GMDVVALT.[3].MRTISIAHRG.[14]. 290
gi 120609006 256 GTRAVPLR.[3].AREISVAFRR.[15]. 291
gi 152966772 263 GVRSVPVV.[4].TRTIYVVVRK.[15]. 299
gi 134096962 256 RIVGRRVR.[3].HRSISAYVLR.[15]. 291
gi 134102712 256 GTVHRPLA.[4].FRRIHAVVPA.[15]. 292
gi 226364736 256 DVRSIPLA.[4].QRRILIAHLT.[15]. 292
gi 119963461 257 NVVRLRIT.[5].SRRIVAAVRR.[15]. 294
```

Fig. S2.

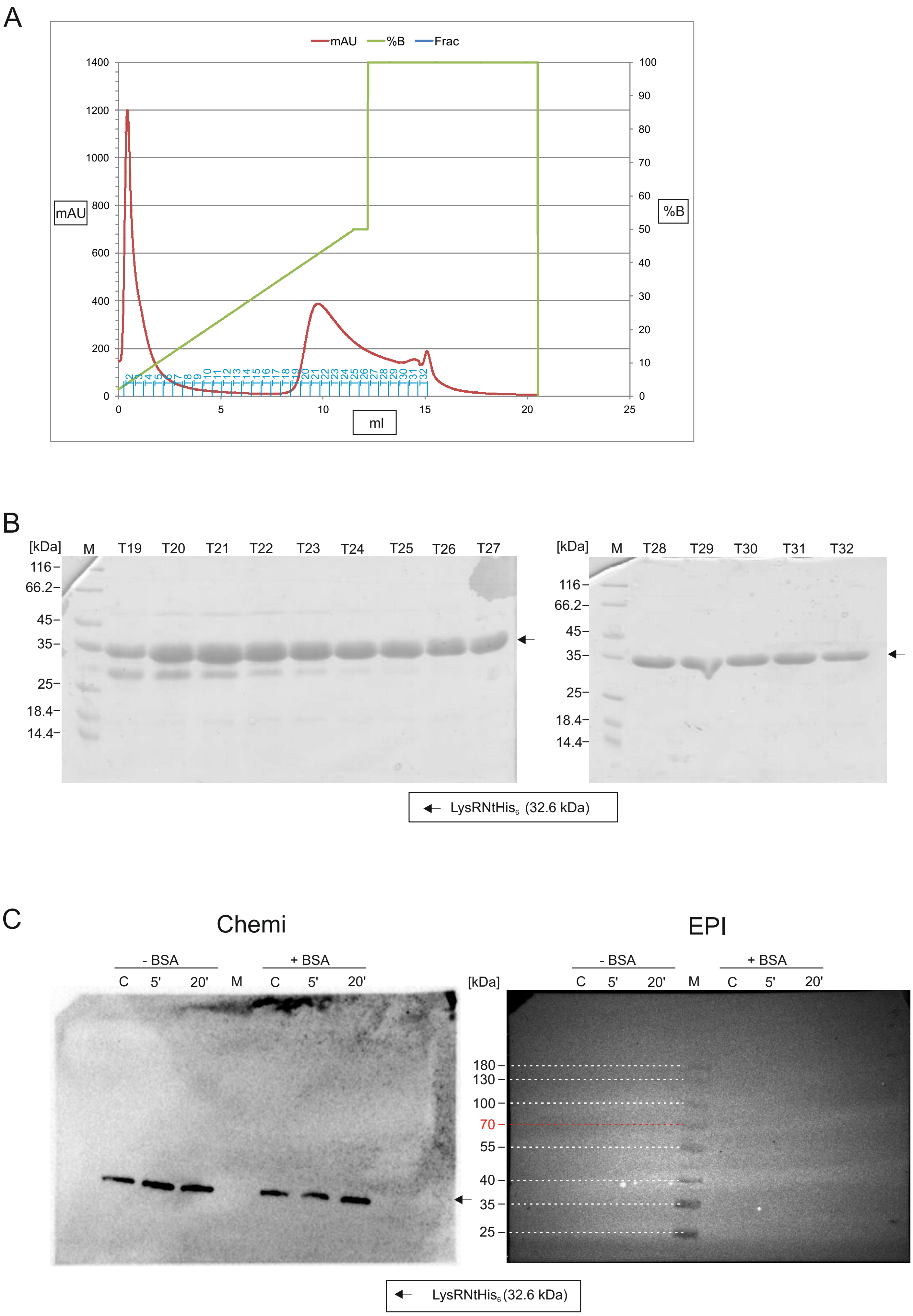
A
mAU
%B
Frac
1400
1200
1000
800
600
400
200
0
100
90
80
70
60
50
40
30
20
10
0
0
5
10
15
20
25
ml
B
[kDa]
M
T19
T20
T21
T22
T23
T24
T25
T26
T27
116
66.2
45
35
25
18.4
14.4
[kDa]
M
T28
T29
T30
T31
T32
116
66.2
45
35
25
18.4
14.4
LysRNtHis6 (32.6 kDa)
C
Chemi
EPI
- BSA
+ BSA
C
5'
20'
M
C
5'
20'
[kDa]
180
130
100
70
55
40
35
25
LysRNtHis6 (32.6 kDa)

Fig. S3.

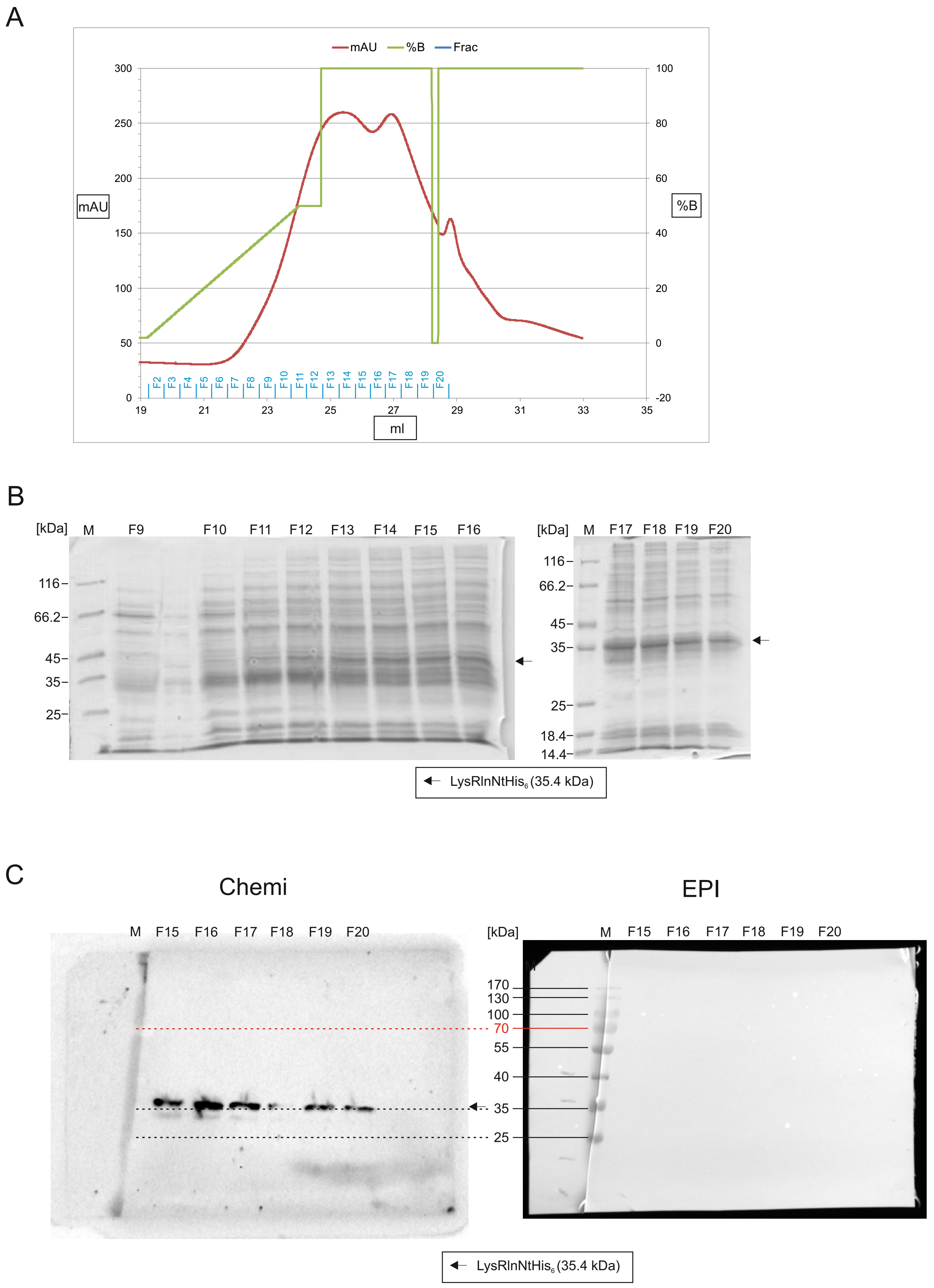
A
mAU
%B
Frac
300
250
200
150
100
50
0
100
80
60
40
20
0
-20
19
21
23
25
27
29
31
33
35
ml
F2
F3
F4
F5
F6
F7
F8
F9
F10
F11
F12
F13
F14
F15
F16
F17
F18
F19
F20
B
[kDa]
M
F9
F10
F11
F12
F13
F14
F15
F16
116
66.2
45
35
25
[kDa]
M
F17
F18
F19
F20
116
66.2
45
35
25
18.4
14.4
LysRlnNtHis6 (35.4 kDa)
C
Chemi
EPI
M
F15
F16
F17
F18
F19
F20
[kDa]
M
F15
F16
F17
F18
F19
F20
170
130
100
70
55
40
35
25
LysRlnNtHis6 (35.4 kDa)

Fig. S4.

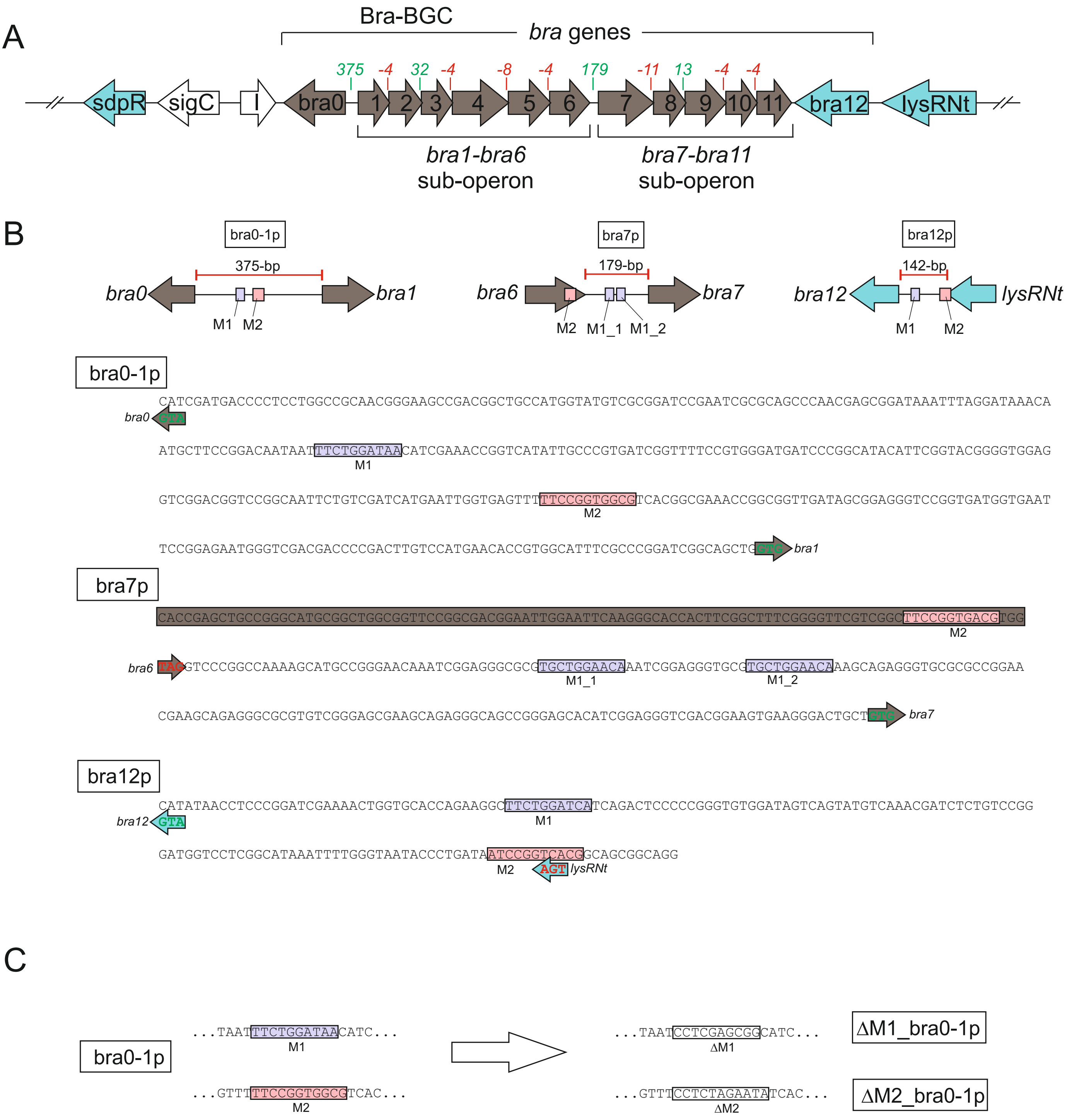
A
Bra-BGC
bra genes
375
-4
32
-4
-8
-4
179
-11
13
-4
-4
sdpR
sigC
I
bra0
1
2
3
4
5
6
7
8
9
10
11
bra12
lysRNt
bra1-bra6
sub-operon
bra7-bra11
sub-operon
B
bra0-1p
375-bp
bra0
bra1
M1
M2
bra7p
179-bp
bra6
bra7
M2
M1_1
M1_2
bra12p
142-bp
bra12
lysRNt
M1
M2
bra0-1p
CATCGATGACCCCTCCTGGCCGCAACGGGAAGCCGACGGCTGCCATGGTATGTCGCGGATCCGAATCGCGCAGCCCAACGAGCGGATAAATTTAGGATAAACA
bra0
GTA
ATGCTTCCGGACAATAATTTCTGGATAACATCGAAACCGGTCATATTGCCCGTGATCGGTTTTCCGTGGGATGATCCCGGCATACATTCGGTACGGGGTGGAG
M1
GTCGGACGGTCCGGCAATTCTGTCGATCATGAATTGGTGAGTTTTTCCGGTGGCGTCACGGCGAAACCGGCGGTTGATAGCGGAGGGTCCGGTGATGGTGAAT
M2
TCCGGAGAATGGGTCGACGACCCCGACTTGTCCATGAACACCGTGGCATTTCGCCCGGATCGGCAGCTGGTG
bra1
bra7p
CACCGAGCTGCCGGGCATGCGGCTGGCGGTTCCGGCGACGGAATTGGAATTCAAGGGCACCACTTCGGCTTTCGGGGTTCGTCGGCTTCCGGTGACGTGG
M2
bra6
TAGGTCCCGGCCAAAAGCATGCCGGGAACAAATCGGAGGGCGCGTGCTGGAACAAATCGGAGGGTGCGTGCTGGAACAAAGCAGAGGGTGCGCGCCGGAA
M1_1
M1_2
CGAAGCAGAGGGCGCGTGTCGGGAGCGAAGCAGAGGGCAGCCGGGAGCACATCGGAGGGTCGACGGAAGTGAAGGGACTGCTGTG
bra7
bra12p
CATATAACCTCCCGGATCGAAAACTGGTGCACCAGAAGGCTTCTGGATCATCAGACTCCCCCGGGTGTGGATAGTCAGTATGTCAAACGATCTCTGTCCGG
M1
bra12
GTA
GATGGTCCTCGGCATAAATTTTGGGTAATACCCTGATAATCCGGTCACGGCAGCGGCAGG
M2
AGT
lysRNt
C
bra0-1p
...TAATTTCTGGATAACATC...
M1
...GTTTTTCCGGTGGCGTCAC...
M2
...TAATCCTCGAGCGGCATC...
ΔM1
...GTTTCCTCTAGAATATCAC...
ΔM2
ΔM1_bra0-1p
ΔM2_bra0-1p

Fig. S5.

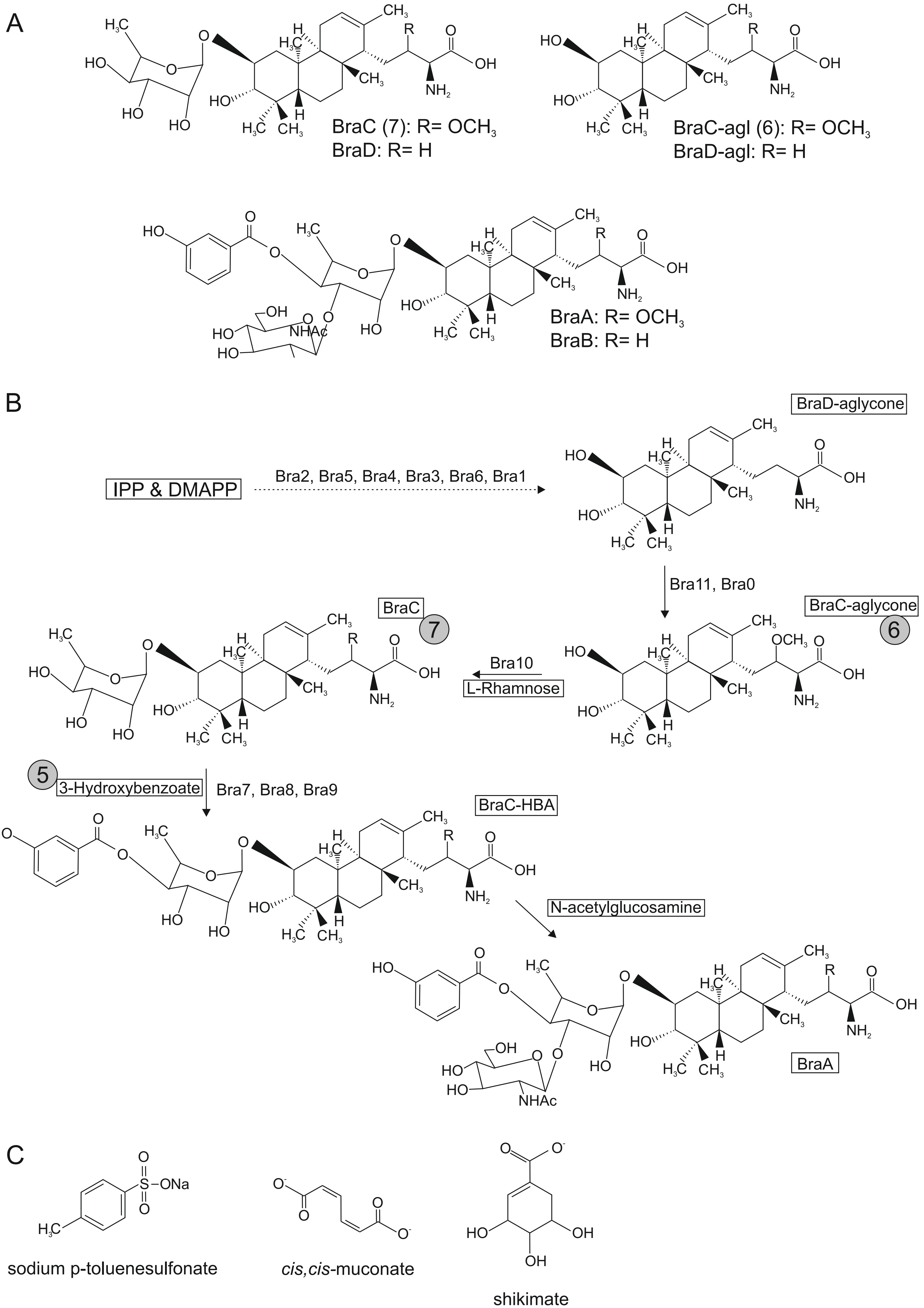
A
BraC (7): R= OCH3
BraD: R= H
BraC-agl (6): R= OCH3
BraD-agl: R= H
BraA: R= OCH3
BraB: R= H
B
BraD-aglycone
IPP & DMAPP
Bra2, Bra5, Bra4, Bra3, Bra6, Bra1
Bra11, Bra0
BraC-aglycone
6
BraC
7
Bra10
L-Rhamnose
5
3-Hydroxybenzoate
Bra7, Bra8, Bra9
BraC-HBA
N-acetylglucosamine
BraA
C
sodium p-toluenesulfonate
cis,cis-muconate
shikimate

Fig. S6.

A

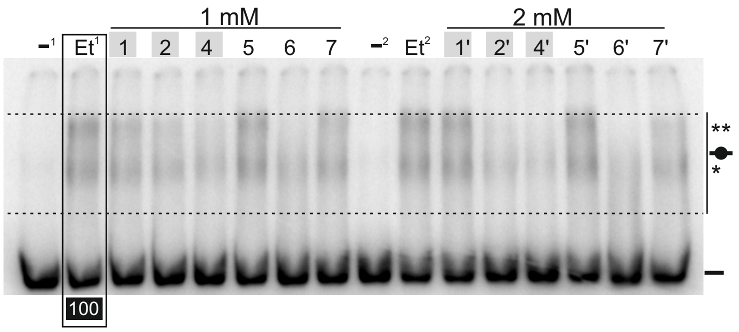

B

cross-linked

sample	-[1]	Et[1]	1	1'	2	2'	4	4'	5	5'	6	6'	7	7'	Et[2]	-[2]
%	5	100	77	86	62	44	47	29	86	84	37	18	69	71	88	5

C

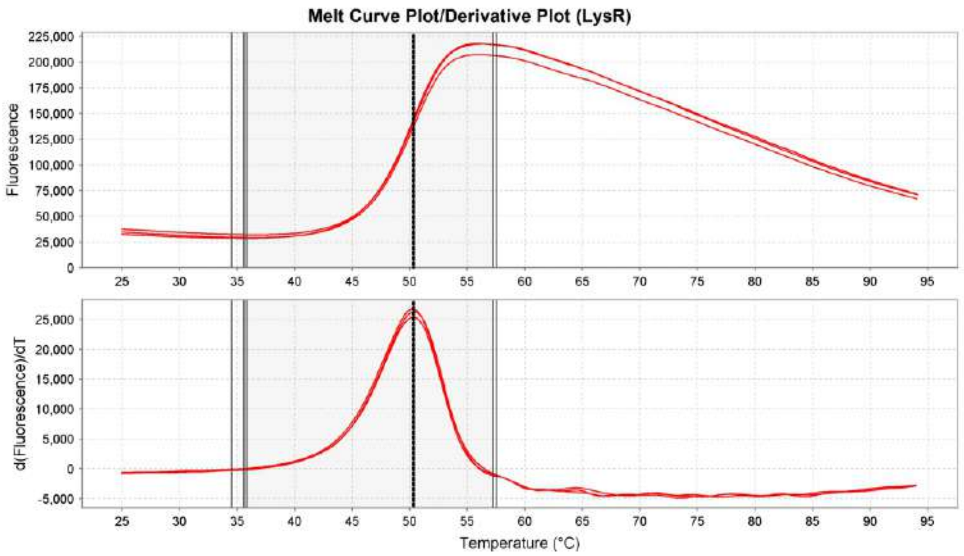

Fig. S7.
